# RNAseq-based gene expression profiling of the *Anopheles funestus* pyrethroid-resistant strain FUMOZ highlights the predominant role of the duplicated *CYP6P9a/b* cytochrome P450s

**DOI:** 10.1093/g3journal/jkab352

**Published:** 2021-10-09

**Authors:** Charles S Wondji, Jack Hearn, Helen Irving, Murielle J Wondji, Gareth Weedall

**Affiliations:** 1 Vector Biology Department, Liverpool School of Tropical Medicine, Liverpool L3 5QA, UK; 2 LSTM Research Unit, Centre for Research in Infectious Diseases (CRID), Yaoundé, P.O. Box 1359, Cameroon; 3 Entomology Unit, International Institute of Tropical Agriculture (IITA), Yaoundé, P.O. Box 2008, Cameroon; 4 School of Natural Sciences and Psychology, Liverpool John Moores University, Liverpool L3 3AF, UK

**Keywords:** malaria, mosquito, *Anopheles funestus*, pyrethroid resistance, metabolic resistance, RNAseq, cytochrome P450s

## Abstract

Insecticide-based interventions, notably long-lasting insecticidal nets, against mosquito vectors of malaria are currently threatened by pyrethroid resistance. Here, we contrasted RNAseq-based gene expression profiling of laboratory-resistant (FUMOZ) and susceptible (FANG) strains of the major malaria vector *Anopheles funestus*. Cytochrome P450 genes were the predominant over-expressed detoxification genes in FUMOZ, with high expression of the duplicated CYP6P9a (fold-change of 82.23 *vs* FANG) and CYP6P9b (FC 11.15). Other over-expressed P450s belonged to the same cluster of P450s corresponding to the resistance to pyrethroid 1 (*rp1*) quantitative trait loci (QTL) on chromosome 2R. Several Epsilon class glutathione S-transferases were also over-expressed in FUMOZ, as was the ATP-binding cassette transporter AFUN019220 (ABCA) which also exhibited between-strain alternative splicing events at exon 7. Significant differences in single-nucleotide polymorphism frequencies between strains occurred in resistance QTLs *rp1* (*CYP6P9a/b* and *CYP6AA1*), *rp2* on chromosome 2L (*CYP6Z1*, *CYP6M7*, and *CYP6Z3*), and *rp3* on chromosome 3R (*CYP9J5*, *CYP9J4*, and *CYP9J3*). Differences were also detected in *CYP4G17 and CYP4G16* genes on the X chromosome, both of which are associated with cuticular resistance in *Anopheles gambiae*. A close analysis of nonsynonymous diversity at the CYP6P9a/b loci revealed a drastic loss of diversity in FUMOZ with only a single polymorphism and 2 haplotypes *vs* 18 substitutions and 8 haplotypes in FANG. By contrast, a lowly expressed cytochrome P450 (CYP4C36) did not exhibit diversity differences between strains. We also detected the known pyrethroid resistance conferring amino acid change N384S in *CYP6P9b*. This study further elucidates the molecular bases of resistance in *An. funestus*, informing strategies to better manage widespread resistance across Africa.

## Introduction

Malaria prevention still heavily relies on insecticide-based interventions including long-lasting insecticidal nets (LLINs) and indoor residual spraying. These tools have contributed massively to the reduction of malaria burden observed since 2000 (Bhatt *et al.* 2015). LLINs have mainly relied on pyrethroid insecticides due to their low toxicity to humans. Unfortunately, major malaria vectors have developed resistance to pyrethroids as a response to the massive scale up of LLINs (Hemingway *et al.* 2016), and also due to ongoing selection from agricultural use of insecticides (WHO 2012). There is increasing evidence that this resistance is jeopardizing the efficacy of LLINs (Mugenzi *et al.* 2019; Weedall *et al.* 2019) and is compromising the success of malaria control programs (Protopopoff *et al.* 2018). It is vital to implement suitable resistance management strategies to prevent the loss of all the gains recently achieved in reducing malaria burdens (Hemingway *et al.* 2016). Understanding the molecular mechanisms of insecticide resistance in major malaria vectors such as *Anopheles funestus*, a major malaria vector in sub-Saharan Africa alongside members of the *Anopheles* *gambiae* complex is, therefore, of great importance for tracking and managing this resistance.

There are primarily two major mechanisms by which insecticide resistance occurs in mosquitoes, namely target-site and metabolic resistance (Hemingway and Ranson 2000; Hemingway *et al.* 2004). Target-site resistance occurs when the mosquito target of an insecticide changes in such a manner as to prevent an insecticide from functioning, for example by reduced binding affinity. Pyrethroid class insecticides target the voltage-gated sodium channel (VGSC) of insects and several mutations of VGSC labeled “knock down” or *kdr* mutations have been identified in several mosquito species (Hemingway and Ranson 2000; Hemingway *et al.* 2004). Intriguingly, *kdr* mutations have yet to be seen in *An. funestus* (Irving and Wondji 2017) but are common in other *Anopheles* vectors (Riveron *et al.* 2018a). Metabolic resistance is the rapid destruction or removal of insecticides by resistant *vs* susceptible insects and is mediated by more efficient and/or higher levels of detoxifying enzymes (Hemingway *et al.* 2004). This mechanism is predominant in *An. funestus* resistance to pyrethroids and occurs through the over-expression of cytochrome P450 class genes (Riveron *et al.* 2018a). Glutathione S-transferases and carboxylesterases can also act to detoxify various insecticides through elevated expression (Riveron *et al.* 2018a).

In recent years, increasing resistance to multiple insecticides has been reported in *An. funestus* species with the first reports emerging from southern Africa (Hargreaves *et al.* 2000; Casimiro *et al.* 2006; Cuamba *et al.* 2010; Riveron *et al.* 2015). Resistance to pyrethroids has been gradually reported in other regions of Africa from including West (Djouaka *et al.* 2011, 2016; Samb *et al.* 2016), Central (Menze *et al.* 2016, 2018; Riveron *et al.* 2018b), and East (Morgan *et al.* 2010; Kawada *et al.* 2011; Mulamba *et al.* 2014), which has spurred efforts to generate new insecticides with different modes of action to maintain control of pyrethroid-resistant mosquitoes (Hemingway *et al.* 2016). However, it is vital to avoid potential cross-resistance between forthcoming novel insecticides and pyrethroids. Hence, it is important to use laboratory pyrethroid resistance strains of malaria vectors which have been well characterized for understanding the molecular basis of resistance, especially fully susceptible laboratory-maintained strains as natural populations are no longer susceptible to pyrethroids.

Two laboratory strains of *An. funestus* have been successfully established by the Vector Control Reference Laboratory at the National Institute for communicable diseases in South Africa. A pyrethroid-resistant *An. funestus* strain named FUMOZ was established from mosquitoes collected in southern Mozambique in 2000 (Hunt *et al.* 2005); this strain is also resistant to carbamates. The pyrethroid-susceptible strain FANG was established from *An. funestus* mosquitoes collected from southern Angola in 2002 and is fully susceptible to all insecticides. Quantitative trait loci (QTL) approaches demonstrated that pyrethroid resistance in FUMOZ was driven by several cytochrome P450 associated loci, among which one locus named resistance to pyrethroids (*rp1*) locus was predominantly driving resistance (Wondji *et al.* 2007, 2009). However, the genome-wide transcription profile associated with resistance specific to the FUMOZ lab strain has not been characterized. Such elucidation will help to identify potential drivers of a cross-resistance between pyrethroids and carbamates resistance. It could also help to establish a correlation between QTLs and transcription profile (Wondji *et al.* 2007, 2009). Furthermore, a full transcriptomic profiling of the FUMOZ-resistant strain will serve as a useful reference when screening novel insecticide candidates. Such a profile will help explain underlying molecular drivers of any observed cross-resistance, and thus help select novel insecticides and combinations thereof that minimize the risk of quick resistance development.

Here, using an RNAseq-based transcription analysis, we characterized the gene expression profiles of two laboratory-adapted colonies of *An. funestus*: one selected for resistance to multiple insecticides and the other fully susceptible. This study has revealed a strong association between the over-expression of major resistance genes and the presence of signatures of selective sweep as the duplicated P450 genes *CYP6P9a and CYP6P9b* are massively over-expressed in FUMOZ while exhibiting a drastically reduced diversity compared to the susceptible FANG strain.

## Materials and methods

### Mosquito rearing, RNA extraction, and sequencing

Two *An. funestus* laboratory colonies were used in this study. The FANG colony is a fully insecticide susceptible colony derived from Angola (Hunt *et al.* 2005). The FUMOZ colony is a multi-insecticide resistant colony derived from southern Mozambique, with additional selection for insecticide resistance (Hunt *et al.* 2005). Colonies were maintained in insectaries at the Liverpool School of Tropical Medicine at 28°C, 80% humidity, and a 12 h light:dark cycle. Larvae were grown in mineral water in plastic trays and fed daily with ground TetraMin fish food. Adults were kept in 30 × 30 cm cages and provided with 10% sucrose. Adults were arm-fed to allow females to lay eggs.

Total RNA was extracted from pools of 10 individual 3-day-old non-blood fed adult female mosquitoes using the Arcturus PicoPure RNA isolation kit (Life Technologies, Carlsbad, CA, USA). In this study, four pools of FUMOZ RNA were sequenced and one pool of FANG RNA, alongside three FANG RNAseq libraries from a previous study (Weedall *et al.* 2015). Methods used for RNA extraction, ribosomal RNA-depletion, library preparation, and sequencing were described previously (Weedall *et al.* 2015, 2019). Library preparation and sequencing were undertaken by the Centre for Genomic Research (CGR), University of Liverpool.

### Functional annotation improvement of gene set AfunF3.1

Our analysis used the chromosome-scale *An.* *funestus* FUMOZ colony reference genome assembly AfunF3 and annotation gene set AfunF3.1 [downloaded from https://www.vectorbase.org/ (last accessed date: 11/10/2021) June 25, 2019 (Giraldo-Calderon *et al.* 2015)] (Ghurye *et al.* 2019, preprint). Gene set AfunF3.1 contains many putative genes with no functional descriptions. To aid interpretation of our results, all AfunF3.1 predicted transcripts were used for similarity-based functional annotation assignment using Blast2Go (Conesa *et al.* 2005) using the nonredundant (nr) protein database downloaded from NCBI. Blast (BLASTx) searches against the nonredundant (nr) protein database and InterProScan searches of the InterPro protein signature databases were carried out to further annotate the *An. funestus* protein-coding genes.

### Analysis of RNAseq data

De-multiplexed fastq files were trimmed to remove Illumina adapter sequences (minimum 3 bp match at the 3′ end) using Cutadapt version 1.2.1 (Martin 2011), and low-quality bases trimmed, using Sickle version 1.200 (Joshi and Fass 2011), with a minimum window quality score of 20. After trimming, reads shorter than 10 bp were removed. If both reads from a pair passed this filter, each was included in a forward or reverse reads file. If only one of a read pair passed this filter, it was place in an unpaired reads file.

Strand NGS software, version 3.4 (Strand Life Sciences, Bangalore, India) was used for data analysis. Trimmed R1/R2 read pairs were aligned to the reference sequence (Afun 3.1) using the option ‘transcriptome and genome together’ (with novel splices) which aligns reads to both transcriptome and genome to find the best matches. Reads that remain unaligned after the first-round are then aligned by considering novel splices across known exons and/or novel candidate exons. A custom ‘build’ was generated for *An. funestus* genome in Strand NGS following the program’s instructions. The best matches for a read were reported if they satisfied the following criteria: a minimum percent identity of 90, a maximum percent gaps between matches of 5, a maximum number of novel splices of 2, a minimum match length of 25, a maximum number of matches to be reported for each read equal to 1, and ignoring reads with matches more than 5 matches. Further quality trimming was performed by removing 3′ ends with average base quality <10.

Strand NGS was used to perform RNA quantification, which reports raw counts per gene by counting the total number of reads that map to each gene and exon in the *An. funestus* genome. Raw counts were normalized using DESeq’s inbuilt method (Anders and Huber 2010) which accounts for differences in the total number of reads between samples.

Differential gene expression analysis was carried out using DESeq (Anders and Huber 2010) as implemented in Strand NGS. Two conditions (FUMOZ and FANG, each with four biological replicates) were contrasted. The estimated log_2_ fold-change (FC) for each gene was tested using a moderated *t*-test which is a modification of the unpaired *t*-test (Smyth 2004). *P*-values were adjusted for multiple testing using the false discovery rate (FDR) approach of Storey with bootstrapping (*q*-value). Differentially expressed genes were defined as those with an FDR-adjusted *P*-value <5% and FC ≥2.

Gene ontology (GO) enrichment analysis was carried out on differentially expressed gene sets using Strand NGS. A more detailed analysis was performed for detoxification genes of interest over-expressed in FUMOZ to detect sets of transcripts which are similarly expressed and thus potentially acting together in conferring resistance to FUMOZ. This was performed by selecting the list of transcripts significantly over-expressed in FUMOZ followed by the selection of the transcript of interest and then selecting the similarity metric with a minimum set at 0.5 using Strand NGS. Altogether, this option allows to find transcripts, in a specified list of transcripts (such as those upregulated), whose expression profile matches that of the transcript of interest.

### Analysis of alternative splicing 

Strand NGS identifies differentially spliced genes based on change in transcript proportions across conditions which here is between FANG (susceptible) and FUMOZ (resistant). Here, a threshold of 0.25 was set to detect genes for which the transcript proportion varies between FUMOZ and FANG in which case the gene is termed differentially spliced. The process of determining transcript proportions takes reads which fall within the boundaries of a gene but outside the exon boundaries of any of the associated transcripts and ascribes these reads to an “unknown” transcript. Based on the fraction of reads that get ascribed to this “unknown” transcript, this “unknown” transcript gets a proportion much like any known transcript does. This proportion is checked for variation across conditions exactly as described above. If of all transcripts for the gene, it is the “unknown” transcript that shows the maximum change across conditions Strand NGS labels this gene as “novel”.

### Quantitative reverse transcriptase PCR

RNAseq expression patterns of some of the most over-expressed detoxification genes were validated by quantitative reverse transcriptase PCR (qRT-PCR), including *CYP6P9a* (AFUN015792), *CYP6P9b* (AFUN015889), *GSTe2* (AFUN015809), *CYP6P5* (AFUN015888), *CYP325A* (AFUN015966), and *CYP6AA1* (AFUN015786). Total RNA was extracted from 3 batches of 10 mosquitoes for each strain using the picopure RNA extraction kit, as previously described (Riveron *et al.* 2013). The primers are listed in Supplementary Table S1. Briefly, 1 µg of total RNA was used for cDNA synthesis using Superscript III (Invitrogen) with oligo-dT20 and RNase H according to the manufacturer’s instructions. The qRT-PCR amplification was performed following standard protocol (Kwiatkowska *et al.* 2013; Riveron *et al.* 2013) after establishing the standard curves for each gene to assess PCR efficiency and quantitative differences between samples using serial dilution. The relative expression of each gene was calculated according to the 2^−^^ΔΔCT^ method (Schmittgen and Livak 2008) and compared between the two strains after normalization with the housekeeping genes ribosomal protein S7 (*RSP7*; AFUN007153) and actin 5C (AFUN006819).

### Detection of single-nucleotide polymorphism

The four FUMOZ and four FANG replicates were combined into a single sample to detect the relevant polymorphisms. All variant types [single-nucleotide polymorphisms (SNPs), multiple nucleotide polymorphisms (MNPs), and indels or MNPs] were detected by comparing against the FUMOZ genome using the MAQ independent model implemented in Strand NGS. Variant calling parameters thresholds were a phred-scaled confidence score cutoff of 50 or above. This score represents the confidence in the variant call. Other criteria include a coverage greater than 10, ignoring homopolymer of 10, or higher as well as the locations adjacent to them. A filtering step was performed as implemented in Strand NGS through the “HomPolyfilter” flag which removes indels calls with <25% supporting reads in homopolymer stretches and “IndelSR20” which removes indels with <20% of supporting reads. An SNP multi-sample report was generated which included the allele frequency in the population based on the number of supporting reads for each variant, the zygosity, genotype, score, and other attributes such as % of supporting reads and variants. For each variant, its SNP effect (nonsynonymous, synonymous, 5′ UTR, 3′ UTR etc.) and the affected transcripts were predicted using the gff3 transcript annotation (Afun3.1).

### Detection of significant SNPs

To detect SNPs significantly associated with pyrethroid resistance, we took two approaches. Firstly, we used a stringent differential allele frequency-based approach where a variant was considered significant in relation to pyrethroid resistance if the supporting read % range was 0–10% in all four FUMOZ samples, but 90–100% in all the four FANG samples. This is because the AFUN3.1 genome was generated from FUMOZ strain mosquitoes meaning resistance-conferring alleles are present in the reference sequence.

The second approach assessed the significant association between each variant with permethrin resistance by estimating the unpaired *t*-test of each variant between both and the –Log_10_ of the resulting *P*-value used to generate a Manhattan plot for each chromosome. A cutoff of minimum of two samples for plotted SNPs was applied. Furthermore, analysis of the 2R chromosome region spanning the rp1 QTL was comparatively performed between both strains to detect signature of selection in the resistant FUMOZ strain. Briefly, the percentage of supporting reads at each variant was averaged between the four samples of each strain and plotted across the 120 kb region.

### Polymorphism of candidate resistance genes *CYP6P9a* and *CYP6P9b*

Particular attention was given to the genes *CYP6P9a and CYP6P9b* which result from a fixed duplication of CYP6P9 in *An. funestus* compared to other *Anopheles* malaria vector species (Wondji *et al.* 2009). Polymorphisms in these genes were comparatively assessed between the two strains by focusing primarily on nonsynonymous SNPs. Each replicate was arbitrarily considered as an individual to facilitate the analysis. Data from the SNP multi-sample report were used to retrieve the polymorphisms observed in each sample and this information was transferred to the full length of each gene retrieved from Vectorbase. Bioedit was used to input various polymorphisms using an ambiguous letter to indicate heterozygote positions. Haplotype reconstruction and polymorphism analysis were performed in DnaSPv5.10 (Librado and Rozas 2009), and MEGA X (Kumar *et al.* 2018) was used to construct the maximum likelihood phylogenetic tree for each gene.

## Results

### Differentially expressed genes

A range of 43–65 million reads were obtained for each of the eight samples sequenced and successfully aligned to the *An. funestus* genome Afun 3.1 as illustrated by the quality metrics (Supplementary Table S2).

#### Genes over-expressed in FUMOZ

**Figure 1:**
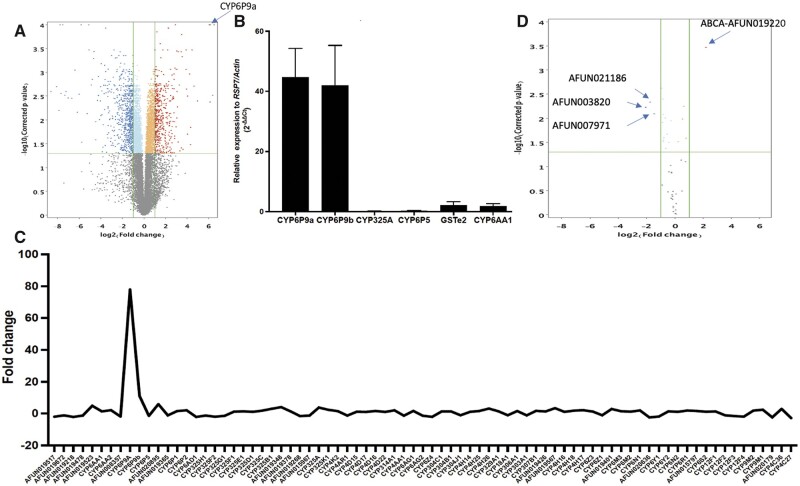
Comparative transcription profiles of FUMOZ and FANG strains: (A) Volcano plot of differential gene expression between pyrethroid-resistant FUMOZ and susceptible FANG. (B) Gene expression pattern of all cytochrome P450 genes on chromosome 2 highlighting the massive expression of *CYP6P9a/b* in FUMOZ. (C) qRT-PCR comparison of the expression profile of major detoxification genes associated with pyrethroid resistance between FUMOZ and FANG. The data are shown as mean + SEM (*n* = 3). (D) Volcano plot showing the differential expression of alternative spliced genes highlighting the ABCA AFUN019220 as the single over-expressed and spliced gene in FUMOZ.

Seven hundred seventeen transcripts were over-expressed in FUMOZ and 1008 downregulated compared to FANG (Figure 1A). Analysis of the list of transcripts upregulated in FUMOZ revealed that among the most over-expressed with FC greater than 10 or more, the transcript with the highest FC at 83 is the AFUN019794 which is an Integrase catalytic domain-containing protein. This list of highly over-expressed genes in FUMOZ also contains the duplicated cytochrome P450s *CYP6P9a* (FC 82.23) and *CYP6P9b* (FC 11.15). Other genes belong to serine proteases and others are transposon-related genes (Supplementary Table S3).

Overall, the list of upregulated genes is dominated by genes belonging to several gene families associated with insecticide resistance including detoxification genes such as cytochrome P450s, glutathione-S-transferases (GSTs), and carboxylesterases (Table 1; Supplementary Figure S1A). Among P450s, the duplicated *CYP6P9a and CYP6P9b* are by far the most over-expressed. The predominance of *CYP6P9a/b* is further evident by the fact that no other P450 gene has FC >6 and only three other P450s present expression of >3 notably *CYP6P4a* (FC 5.9), *CYP325J1* (FC 4.9), and *CYP4C36*. The other P450s have low expression with FC <3 including *CYP6M4*, *CYP6AA2*, and *CYP6P2* also on the 2R chromosome on the same cluster as *CYP6P9a/b*.

**Table 1:** List of most upregulated detoxification genes in FUMOZ strain

Gene ID	*P*	FC	Log FC	Gene symbol	FUMOZ reads	FANG reads	Description
**Cytochrome P450**							
AFUN015792	0.0005	78.0	6.3	CYP6P9a	84,176.5	822.6	Cytochrome P450
AFUN015889	0.0015	11.1	3.5	CYP6P9b	11,906.6	744.2	Cytochrome P450
AFUN020895	0.0065	5.9	2.6	CYP6P4a	1,895.4	255.5	Cytochrome P450
AFUN019523	0.0466	4.9	2.3	CYP325J1	19.6	3.4	Cytochrome P450
AFUN006135	0.0049	3.0	1.6	CYP4C36	987.1	261.9	Cytochrome P450
AFUN019401	0.0407	2.9	1.5	CYP6M4	2,115.5	731.1	Cytochrome P450
AFUN021097	0.0491	2.5	1.3	CYP4H24	2,613.8	1,057.2	Cytochrome P450
AFUN015938	0.0042	2.5	1.3	CYP9M1	1,962.9	585.2	Cytochrome P450
AFUN007549	0.0018	2.4	1.3	CYP9K1	3,991.8	1,049.2	Cytochrome P450
AFUN015785	0.0076	2.1	1.1	CYP6AA2	776.2	254.4	Cytochrome P450
AFUN015801	0.0015	2.1	1.1	CYP6P2	2,106.3	718.8	Cytochrome P450
AFUN002978	0.0073	2.1	1.0	CYP314A1	642.4	219.3	Cytochrome P450
**Glutathione S-transferases (GSTs)**							
AFUN022201	0.0081	11.8	3.6	AFUN022201	54.3	5.0	Glutathione peroxidase
AFUN016008	0.0072	4.0	2.0	GSTE6	605.7	125.5	Glutathione S-transferase
AFUN015808	0.0038	3.6	1.8	GSTE3	3,351.1	772.5	Glutathione S-transferase
AFUN015809	0.0013	3.3	1.7	GSTE2	2,415.9	491.8	Glutathione S-transferase
AFUN015839	0.0034	3.1	1.6	GSTD3	1,298.3	332.6	Glutathione S-transferase
AFUN015811	0.0032	2.5	1.3	GSTE5	1,185.9	332.4	Glutathione S-transferase
AFUN016010	0.0031	2.4	1.3	GSTD1	13,199.4	4,246.1	Glutathione S-transferase
AFUN007291	0.0217	2.4	1.2	GSTT2	660.4	238.0	Glutathione S-transferase
**Carboxylesterases**							
AFUN016265	0.0177	7.2	2.9	AFUN016265	387.8	51.8	Carboxylesterase
AFUN016264	0.0119	2.4	1.3	AFUN016264	240.6	73.6	Carboxylesterase
AFUN000374	0.0037	2.2	1.2	AFUN000374	2,557.4	846.9	Carboxylesterase
**Other detox genes**							
AFUN004354	0.0103	2.1	1.1	AFUN004354	1,005.3	317.5	UDP-glucuronosyltransferase
AFUN020670	0.0041	2.4	1.3	AFUN020670	995.7	247.5	Xanthine dehydrogenase
AFUN008239	0.0149	2.7	1.5	AFUN008239	830.9	225.8	Sulfotransferase
AFUN016207	0.0047	4.6	2.2	AFUN016207	699.0	162.8	Sulfotransferase
AFUN016205	0.0246	2.8	1.5	AFUN016205	1,290.1	398.8	Sulfotransferase
AFUN016210	0.0054	2.4	1.3	AFUN016210	297.2	91.2	Sulfotransferase
**Transporters**							
AFUN019220	0.0020	4.7	2.2	AFUN019220	4,442.5	676.7	ATP-binding cassette transporter
AFUN016412	0.0105	2.5	1.3	AFUN016412	5,425.0	1,795.5	Solute carrier family 15 member
AFUN000622	0.0099	3.8	1.9	AFUN000622	1,350.9	235.3	Solute carrier family 23 (
AFUN015826	0.0013	2.1	1.0	AFUN015826	1,479.5	496.5	Solute carrier family 7
AFUN021547	0.0087	2.8	1.5	AFUN021547	513.9	144.3	Sugar transporter SWEET
AFUN021546	0.0200	3.1	1.6	AFUN021546	1,490.9	362.5	Sugar transporter SWEET1
**Mitochondrial/redox**							
AFUN010502	0.0007	8.2	3.0	AFUN010502	4,959.9	534.9	ATP synthase subunit d, mitochondrial
AFUN011500	0.0013	2.7	1.4	AFUN011500	2,297.9	686.9	Sulfite oxidase
AFUN000619	0.0025	2.4	1.3	GRX2	768.9	303.6	Glutaredoxin
AFUN011515	0.0063	2.0	1.0	TPX4	3,567.4	1,179.5	Thioredoxin peroxidase 4
AFUN019604	0.0042	2.7	1.4	AFUN019604	30.6	10.7	Glutaredoxin-3
AFUN001952	0.0049	4.0	2.0	AFUN001952	1,159.8	267.4	Uncoupling protein 2, mitochondrial
**Proteases and others**							
AFUN016100	0.0059	2.1	1.1	AFUN016100	2,012.7	648.1	Alpha-amylase
AFUN019614	0.0039	3.0	1.6	AFUN019614	1,413.7	323.6	Ankyrin repeat
AFUN004002	0.0021	2.7	1.4	AFUN004002	17,374.3	4,616.8	Argininosuccinate lyase
AFUN019821	0.0126	2.0	1.0	AFUN019821	905.3	334.1	Carboxypeptidase A
AFUN021240	0.0013	17.8	4.2	AFUN021240	1,050.2	46.2	Carboxypeptidase Q
AFUN021239	0.0079	11.5	3.5	AFUN021239	807.5	75.4	Carboxypeptidase Q
AFUN022011	0.0200	3.2	1.7	AFUN022011	50,117.5	15,637.6	Chymotrypsin
AFUN007012	0.0442	5.0	2.3	AFUN007012	12.3	4.8	Chymotrypsin-like protease
AFUN016591	0.0043	7.1	2.8	AFUN016591	24,258.5	2,746.8	Trypsin
AFUN016449	0.0222	2.3	1.2	AFUN016449	3,458.1	1,127.3	Trypsin
AFUN020614	0.0052	3.7	1.9	AFUN020614	3,655.9	848.5	Trypsin-6
AFUN022010	0.0131	4.3	2.1	AFUN022010	101,129.8	20,343.8	Serine protease; Trypsin-1
AFUN020946	0.0192	23.3	4.5	AFUN020946	153.9	10.8	Serine-type endopeptidase -Chymotrypsin-2
**Cuticular proteins**							
AFUN011620	0.0154	7.8	3.0	AFUN011620	92.4	11.7	Cuticle protein
AFUN009937	0.0101	3.9	2.0	AFUN009937	5.0	1.4	Cuticular protein RR-1 family
AFUN006418	0.0235	2.8	1.5	AFUN006418	282.1	72.0	Cuticular protein RR-2 family
AFUN020923	0.0259	15.7	4.0	AFUN020923	57.6	3.1	Cuticular protein RR-2 family
AFUN021592	0.0155	2.4	1.3	AFUN021592	194.0	54.1	Larval cuticle protein LCP-30
**Gustatory/odorant receptors**							
AFUN015936	0.0107	5.5	2.5	Gr20	731.4	124.2	Gustatory receptor
AFUN015933	0.0092	2.7	1.4	Gr45	24.4	7.7	Gustatory receptor
AFUN008090	0.0089	2.7	1.4	Or36	19.9	8.1	Odorant receptor
AFUN008522	0.0276	2.2	1.2	Or39	63.3	22.0	Odorant receptor
AFUN018655	0.0028	8.2	3.0	AFUN018655	13.0	0.4	Odorant receptor
**MicroRNA**							
AFUN017296	0.0002	4.3	2.1	mir-279	4.9	0.8	MicroRNA mir-279
AFUN017073	0.0403	2.9	1.5	mir-33	4.5	1.1	MicroRNA mir-33
**Transposon**							
AFUN019794	0.0003	83.2	6.4	AFUN019794	67,882.0	520.4	BEL12_AG transposon polyprotein
AFUN019813	0.0053	22.9	4.5	AFUN019813	4,612.4	121.4	Retrovirus-related Pol polyprotein from transposon TNT 1-94
**Transcription factors**							
AFUN002438	0.0039	4.2	2.1	AFUN002438	446.7	102.3	Transcription elongation factor 1 homolog
AFUN019110	0.0018	2.9	1.5	AFUN019110	631.4	183.8	Transcription factor Adf-1
AFUN021815	0.0042	2.2	1.1	AFUN021815	422.6	152.5	Transcription factor grauzone
AFUN021263	0.0038	3.6	1.9	AFUN021263	425.8	93.9	Transcription initiation factor TFIID subunit
AFUN011301	0.0040	2.6	1.4	AFUN011301	1,434.7	388.1	Transcription initiation factor TFIID subunit 12
AFUN011301	0.0040	2.6	1.4	AFUN011301	1,434.7	388.1	Transcription initiation factor TFIID subunit 12
AFUN021364	0.0370	2.1	1.1	AFUN021364	408.3	155.2	Transcription initiation factor TFIIH subunit

Description, gene annotation; FC, fold-change; FUMOZ/FANG reads, total read pairs across replicates; Log FC, log_2_ fold-change; *P*, FDR-adjusted *P*-value.

Overexpressed GSTs include four genes from the epsilon class, all with moderate FCs notably *GSTe2* (FC 3.3), *GSTe3* (FC 3.6), *GSTe5* (FC 2.5), and *GSTe6* (FC 4.0). Other GSTs include *GSTD3* (FC 3.1), *GSTD1* (FC 2.4), and *GSTT2* (FC 2.4). We also noted that a glutathione peroxidase also exhibits a high FC (FC 11.8) in FUMOZ although the overall number of reads was low (Table 1; Supplementary Figure S1B).

Three carboxylesterases are also over-expressed with AFUN016265 showing an FC of 7.2, an ortholog of AGAP001722 in *An. gambiae*, whereas the other two have FC <3 (AFUN16264 and AFUN000374, ortholog of AGAP006729 in *An. gambiae*).

Other detoxification genes over-expressed also have low FCs including an UDP-glucuronosyltransferase (AFUN004354; FC 2.1), a xanthine dehydrogenase (AFUN020670; FC 2.4), and four sulfotransferases (Table 1; Supplementary Figure S1C).

A group of transport-related genes are also over-expressed in FUMOZ among which the ABC transporter AFUN019220 (FC 4.7) and three solute carrier proteins and two sugar transporters with moderate FC (Table 1). Another set of genes over-expressed belong to mitochondrial redox group along with the ATP synthase subunit d (AFUN010502) is the most highly expressed (FC 8.2) as well as two glutaredoxins and thioredoxin peroxidase 4. A group of genes belonging to proteases are also over-expressed including trypsin, chymotrypsin, two carboxypeptidases Q [AFUN021240 (FC 17.8) and AFUN021239 (FC 11.5)], and arginosuccinate lyase (FC 2.7). Other genes families over-expressed include cuticular proteins, gustatory/odorant receptors genes, two microRNAs although with low number of reads (mir-279 and mir-33), and several transcription factors.

#### Genes downregulated in FUMOZ

Of the 1008 genes significantly downregulated in FUMOZ compared to FANG (Figure 1; Supplementary Table S4), the most downregulated gene is the Group XIIB secretory phospholipase A2-like protein with FC of −602.4. However, this list is dominated by several transcripts belonging to heat shock proteins including heat shock protein 70 A1 (AFUN019775), 70B2 (AFUN019570, AFUN019671, AFUN019513, AFUN019467, and AFUN019289). It also includes Thioredoxin peroxidase 4 (AFUN018693; FC 202.4) which is known to be required for the transcriptional responses to oxidative stress (Ross *et al.* 2000). Some detoxification genes were also downregulated in FUMOZ including cytochrome P450, GSTs, and carboxylesterases although none of these have been previously associated with resistance to insecticides (Supplementary Table S4).

#### qRT-PCR

The qRT-PCR performed with some candidate genes was concordant with the results observed with RNAseq with high expression of both *CYP6P9a* (FC 44.7) and *CYP6P9b* (FC 41.9), whereas the other genes had far lower expression level. These includes *GSTe2*, *CYP6P5*, *CYP325A*, and *CYP6AA1* (Figure 1B).

### Gene ontology enrichment

The GO enrichment analysis of the list of 717 transcripts over-expressed in FUMOZ detected 28 enriched GO terms (Supplementary Figure S2A). There was a predominance of terms associated with peptidase activity including GO:0008233 for peptidase activity known to catalyze the hydrolysis of a peptide bond, GO:0004175 for endopeptidase activity known to catalyze the hydrolysis of internal, alpha-peptide bonds in a polypeptide chain, or GO:0008237 for metallopeptidase activity. Another group of GO terms was related to transport including GO:0005215 for transporter activity, GO:0055085 for transmembrane activity, or GO:0030001 for metal ion transport which is known to direct the movement of metal ions into or out of a cell or even between cells (Supplementary Figure S2A). Other GO terms are associated with mitochondrial activity including GO:0005743 for mitochondrial inner membrane and GO:0019866 for organelle inner membrane. The other category of GO relates to hydrolase activity notably GO:0016787 known to catalyze the hydrolysis of various bonds and GO:0017171 for serine hydrolase activity.

Analysis of the GO enrichment from the list of downregulated transcripts provided a profile like that of upregulated genes (Supplementary Figure S2B) with similar groups of GO terms observed although with different % of enrichment.

### General expression patterns of major detoxification families using all entities

The entire list of detoxification genes was analyzed in detail to identify clusters of genes co-located on chromosomal regions of the genome. Analysis of the expression patterns of the entire P450 list revealed a peak of expression around *CYP6P9a/b* on 2R chromosome which is very similar in profile to the QTL peak previously detected in FUMOZ for the rp1 (resistance to permethrin-1) locus (Wondji *et al.* 2007, 2009) (Figure 1C). No other major peak was detected for P450s either on chromosome 3 or X (Supplementary Figure S3, A and B). No major peak was also observed for GSTs (Supplementary Figure S3C), ABC transporters (Supplementary Figure S3D), and carboxylesterases (Supplementary Figure S3E) with all transcripts exhibiting FC <5.

### Alternative splicing

**Figure 2:**
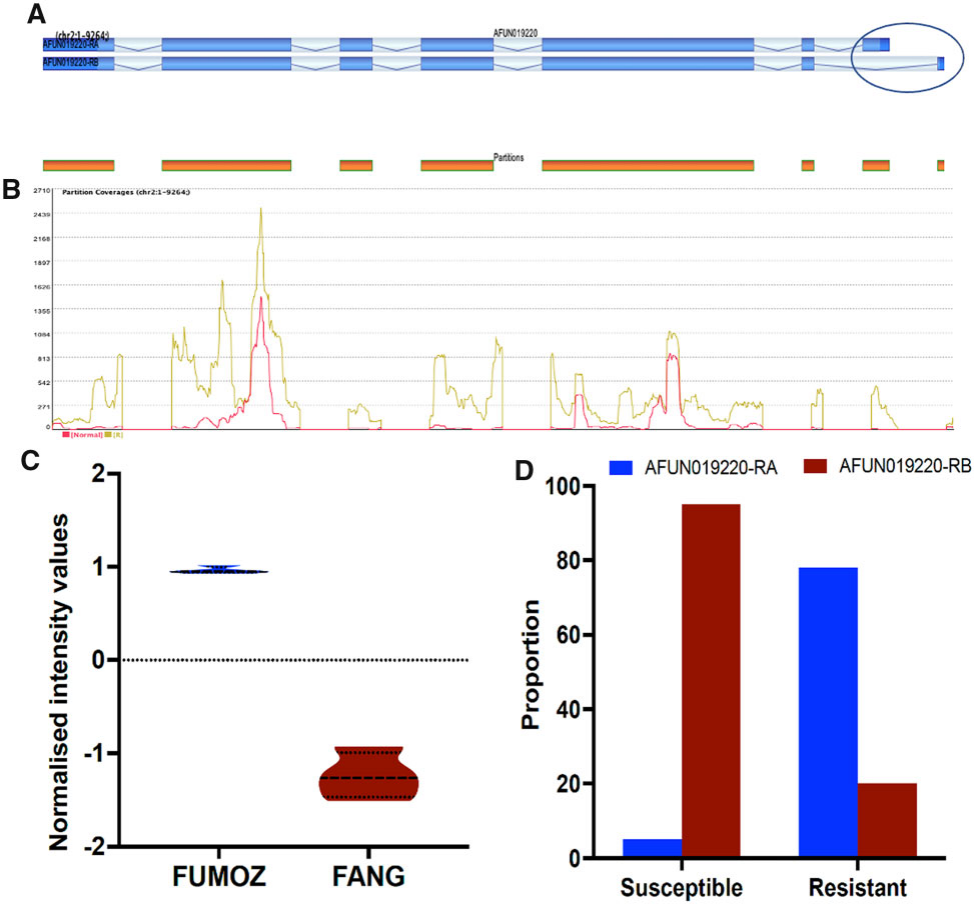
Alternative splicing event at the over-expressed ABC transporter ABCA (AFUN019220). (A) Schematic representation of the transcript organization of this gene with 7 exons for each transcript with last exon 7 having an alternative splice version in AFUN019220-RA and AFUN019220-RB. (B) Comparative read coverage for each exon in each strain. (C) Violin plot showing the greater expression of AFUN019220 in FUMOZ (R) than FANG (Normal). (D) Differential expression of spliced transcripts AFUN019220-RA and -RB in FUMOZ and FANG.

Strand NGS identified 27 genes exhibiting differentially alternative spliced events between FUMOZ and FANG (Figure 1D;Supplementary Table S5). The gene with highest index of alternative splicing was AFUN021186 encoding for a DEAD-box ATP-dependent RNA helicase 30 on chromosome 3 which is over-expressed in the susceptible FANG strain [splicing index (SI) = 0.77]. Among the genes upregulated in FUMOZ, the ABC transporter AFUN019220 had the highest splicing index (0.75). AFUN019220 has 7 exons and two isoforms (see https://vectorbase.org/vectorbase/app/record/gene/AFUN019220; last accessed date: 11/10/2021) with exon 7 exhibiting alternative splicing in this experiment (Figure 2A). Analysis of the partition coverage across the 7 exons supports this difference at exon 7 between the two strains (Figure 2B). Gene-wide expression of this ABC gene is greater in the resistant FUMOZ strain (Violin plot, Figure 2C). A greater proportion of the isoform AFUN019220-RA (78.1%) is observed in the FUMOZ-resistant strain whereas a greater proportion of the other transcript AFUN019220-RB is seen in the susceptible strain (94.6%) (Figure 2D). The other detoxification gene of this list was the carboxylesterase AFUN000373 (SI = 0.33), whereas other genes do not have clear association with resistance mechanisms.

### Patterns of co-expression for major resistance genes

Analysis of the list of transcripts with similar expression to *CYP6P9a* detected 17 transcripts (Supplementary Table S6) with an expression similarity score >0.6 among which the only other detoxification gene was *CYP6P9b* P450 gene (score = 0.63). This could be explained by the very high expression of *CYP6P9a* which thus stands as an outlier compared to other detoxification genes.

*CYP6P9b* in contrast to *CYP6P9a* had similar expression patterns to more genes (Supplementary Table S6). Among detoxification genes, the most similar gene being ABCA (AFUN019220) (SI = 0.77) followed by *CYP6P4a* P450. Other detox genes included the GSTs *GSTe2 and GSTe6* and a carboxylesterase AFUN016265 (Supplementary Table S6). The list of entities with expression similar to *GSTe2* was longer with the closest entity being a xanthine dehydrogenase (SI = 0.91) including six GSTs (with three GST epsilon and three GST Delta class), five P450s (*CYP4C36*, *CYP9K1*, *CYP6N1*, *CYP6P2*, and *CYP9M1*), three sulfotransferases, and a carboxylesterase (AFUN016264). The list of entity with expression similar to ABCA (AFUN019220) had *GSTe2* as the closest (SI = 0.89) with six other GSTs found. It also has five P450 with *CYP4C36* been the closest (Supplementary Table S6).

### SNPs detection: differential significant SNPs in general and for key detoxification gene families

**Figure 3:**
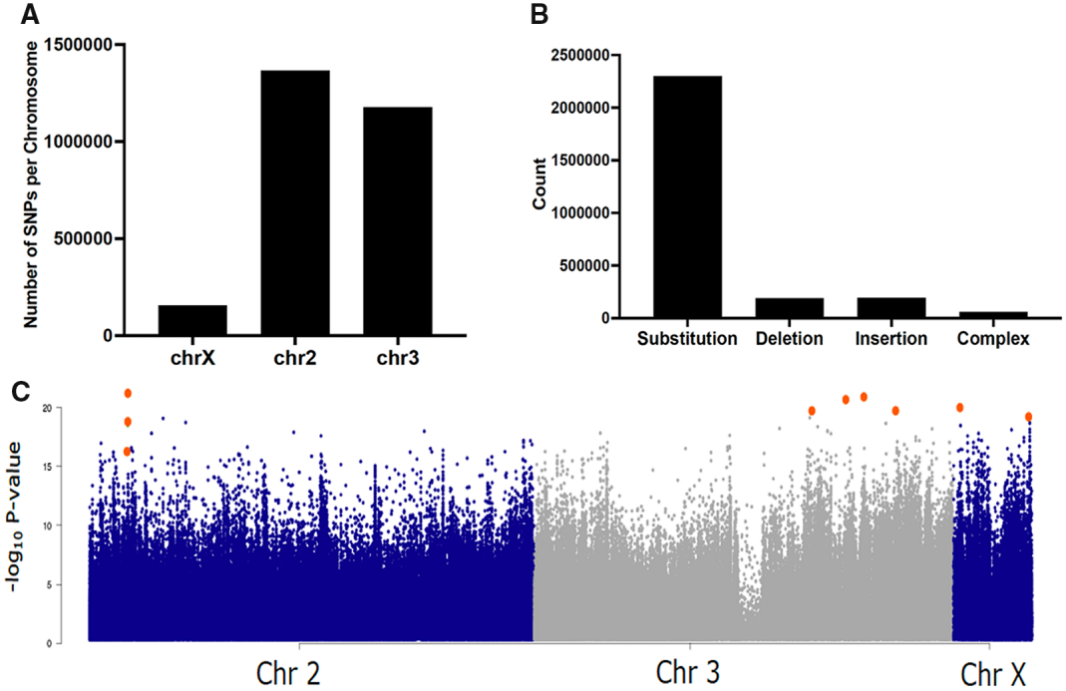
Detection of pyrethroid associated SNPs. (A) Distribution of all detected variants across the 3 chromosomes. (B) Distribution of the variant types detected between strains. (C) Manhattan plot of significant variants associated between FUMOZ and FANG and possibly associated with pyrethroid resistance using an unpaired *t*-test between RNAseq samples of both strains. Large orange circles represent SNPs of interest as discussed in the text and listed in Supplementary Table S9. Figure was created in qqman R package using *T*-test *P*-values as input. Chr 2 = Chromsome 2 (blue), Chr 3 = Chromsome 3 (grey), Chr X = Chromosome X (blue).

A total of 2,793,727 SNPs were detected between FUMOZ and FANG after comparison to the reference AFUN3.1 genome (from FUMOZ strain) and distributed across the three chromosomes from various variant types (Figure 3, A and B). The frequency-based filtering approach, considering only variant with supporting reads percentage between 0–10% for FUMOZ (close to reference genome) and 90–100% for FANG, detected 17,976 variants significantly different between the two strains across 3,599 genes. Of these, 1,596 were nonsynonymous variants and 6,354 synonymous and the remainder occur in 5′ UTR, 3′ UTR, and intron and intergenic regions. When a more stringent selection criteria of consistency in all four samples of each strain was applied, only 2,217 variants were detected including 254 nonsynonymous and 821 synonymous substitutions. A summary of differential SNPs predicted in detoxification genes is presented in Supplementary Table S7. Overall, analysis of SNPs located in detoxification genes highlighted a cluster of SNPs around the genomic region spanning the *rp1* QTL on 2R chromosome belonging to highly over-expressed CYP450s, notably *CYP6P9a and CYP6P9b* (Supplementary Table S7). Other detoxification genes with significant SNPs belonging to cytochrome P450s located in *rp1* included *CYP6AA1*, *CYP6AA2*, *CYP6P4a*, *CYP6P1*, and *CYP6P2* (Supplementary Table S8). A second cluster of SNPs is associated with *rp2* QTL on chromosome 2L including *CYP6Z1*, *CYP6M7*, *CYP6Z3* all previously shown to be conferring pyrethroid resistance (Irving *et al.* 2012; Ibrahim *et al.* 2016). Another cluster of SNPs belong to P450s located on 3R chromosome spanning the previously described *rp3* QTL including *CYP9J5* (previously *CYP9J11*) shown to metabolize pyrethroids (Riveron *et al.* 2017) and CYP9J4 and CYP9J3. Notably, significant SNPs were also detected on chromosome X in *CYP4G17* and *CYP4G16*. Interestingly, significant SNPs also belong to several ABC transporters including the alternatively spliced ABCA (AFUN019220). When looking more specifically at those significant nonsynonymous SNPs in the P450 genes, it was observed that they include a known asparagine to serine change at amino acid 384 (N384S) from *CYP6P9b* (Supplementary Table S8).

The second approach used to detect significant variants using an unpaired *t*-test using percentage of supporting reads, identified sets of SNPs statistically with contrasted between both strains. Several hits were detected spanning the three chromosomes (Figure 3C). The genomic region with the highest *P*-value was in *rp1* region on 2R chromosome. A detailed analysis showed that within this region the most significant SNPs corresponded to *CYP6P9a* gene (*P* = 4.53 × 10^−22^) with two other significant SNPs located in the same gene (Supplementary Table S9; Figure 3). Other highly significant variants were detected on chromosome 3 all in 3′ UTR regions of genes such as cytochrome b5 reductase (AFUN007146), CLIP-domain serine protease (AFUN022249), and an Ecdysone-induced protein 75B (AFUN020438) (Figure 3C). However, none of these genes were differentially expressed between the two strains. Two other SNPs were detected in chromosome X located in 5′ UTR of a cytochrome c oxidase (AFUN019973) and the other with no annotation (Supplementary Table S9; Figure 3C).

**Figure 4:**
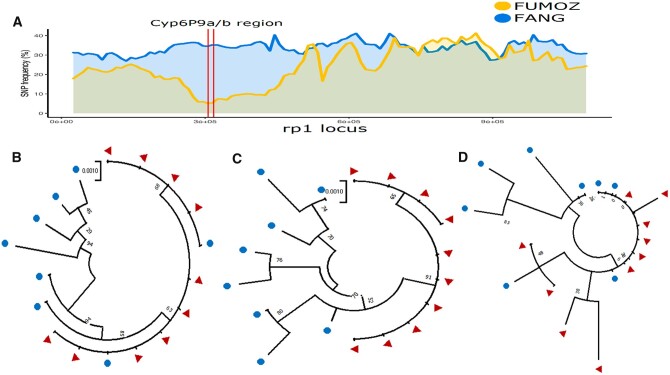
Polymorphism patterns on the genomic regions spanning the *rp1* QTL regions including *CYP6P9a and b*. (A) Comparative SNP frequency along the 2R chromosome region spanning *rp1* showing a marked reduced diversity in FUMOZ notably at the vicinity of the highly over-expressed *CYP6P9a and CYP6P9b* genes. (B) Maximum likelihood phylogenetic tree of *CYP6P9a* sequences generated from RNAseq data between FUMOZ and FANG. (C) Is for *CYP6P9b* and (D) is for *CYP4C36*.

The reduced diversity observed around *rp1* and the consistency of significant SNPs detected in *CYP6P9a and CYP6P9b* led us to further analyze the diversity of both genes (Figure 4A). Another P450 gene *CYP4C36*, upregulated in FUMOZ but at lower FC (3.0), was also analyzed in comparison to the *CYP6P9a/b*. Analysis of nonsynonymous substitutions of *CYP6P9a* revealed a drastic loss of diversity in FUMOZ with only a single polymorphism and two haplotypes *vs* 18 substitutions and eight haplotypes in FANG which was also reflected with lower genetic diversity π in FUMOZ (0.53) than in FANG (7) (Figure 4B; Supplementary Table S10). A similar pattern was also observed for *CYP6P9b* (Figure 4C; Supplementary Table S10). By contrast, *CYP4C36* did not show any difference between strains with similar estimates of genetic diversity for both and we therefore inferred that no selection at this locus (Figure 4D; Supplementary Table S10). Phylogenetic analysis of the three genes further supported this observation with FUMOZ samples clustering together alone for both *CYP6P9a/b* on just two haplotypes, whereas FANG is more diverse (Figure 4). Whereas for *CYP4C36*, samples from both strains are intermingled.

## Discussion

Elucidating mechanisms of resistance to insecticides in malaria vectors is a prerequisite for a successful implementation of resistance management strategies. Here, we took advantage of two phenotypically contrasted laboratory colonies of the major malaria vector *An. funestus* to further decipher the underlying molecular basis of resistance to pyrethroids using RNAseq-based transcription analysis.

### Transcription pattern matches QTL mapping

The transcription profile of both lab strains correlates strongly to previous results obtained through QTL mapping using advanced inter-crossed lines from reciprocal crosses between FUMOZ and FANG (Wondji *et al.* 2007, 2009; Irving *et al.* 2012). Indeed, the most highly overexpressed detoxification gene from this analysis are the duplicated *CYP6P9a* and *CYP6P9b* cytochrome P450s with FCs far greater than other overexpressed genes, previously shown to drive resistance in this strain (Amenya *et al.* 2008; Wondji *et al.* 2009). This is in line with QTL mapping which detected three QTLs associated with pyrethroid resistance with the major one *rp1*, harboring *CYP6P9a and b*, explaining 86% of genetic variance of pyrethroid resistance (Wondji *et al.* 2009). This observation suggests that in a species such as *An. funestus* in which resistance to insecticides is mainly driven by metabolic resistance, combining RNAseq-based transcription analysis to QTL mapping provides a robust methodology for elucidating the molecular basis of resistance. Furthermore, the over-expression of other P450 genes on chromosome 2L and 3R correspond to two other QTL, *rp2 and rp3* which were shown to be minor contributors well in line with the lower expression of these genes. The predominant role of *CYP6P9a/b* in FUMOZ also correlates with the high expression of these genes in wild populations across southern Africa (Riveron *et al.* 2013; Barnes *et al.* 2017a; Weedall *et al.* 2019). This suggests that the colonization of this strain in the laboratory has not significantly altered its genetic background in relation to resistance.

Interestingly, in all three QTL regions, over-expression extends to multiple genes in the gene cluster including in *rp1* where *CYP6P4a*, *CYP6AA2*, and *CYP6P2* were also overexpressed. Such a pattern suggests a similar mechanism of transcriptional regulation applies to the cluster, rather than to an individual gene. While *cis*-regulation of transcription of individual genes likely plays a role (*e.g.*, *CYP6P9a and b* in FUMOZ are much more over-expressed than are other genes in the cluster), other mechanisms such as chromatin remodeling (Clapier *et al.* 2017) around the gene cluster could allow transcriptional co-regulation of all of the genes. As could differential micro-RNA control of gene expression (Feng *et al.* 2018), which we observed for mir-33 and mir-279. Among upregulated P450s, those belonging to the CYP6 family were predominant as reported previously for insecticide-resistant mosquitoes (Djouaka *et al.* 2008; Riveron *et al.* 2018a), although not for *Aedes aegypti* where *CYP9* genes are more abundantly over-expressed (Bariami *et al.* 2012; Ishak *et al.* 2017).

GST genes were also over-expressed, including *GSTe2* and other epsilon class GSTs previously shown to metabolize permethrin, although the 119F resistant allele detected in West/central African populations is absent in FUMOZ (Riveron *et al.* 2013; 2017). Other members of this epsilon class such as *GSTe4 and GSTe5* have been shown to confer pyrethroid resistance either in *An. funestus* (Kouamo *et al.* 2021) and *An. gambiae* (Wilding *et al.* 2015). Altogether, this evidence provides further support a key role for GST enzyme family in pyrethroid resistance.

Besides cytochrome P450 monooxygenases and glutathione S-transferases, a number of genes from other detoxification-associated gene families were also overexpressed in FUMOZ as is often the cases for metabolic resistance (Puinean *et al.* 2010; Bariami *et al.* 2012; Riveron *et al.* 2013). These genes belonging to carboxylestases, sulfotransferases, UDP-glycosyltransferases, and ATP-binding cassette transporter warrant further study to assess their roles in metabolic insecticide resistance which has lagged behind cytochrome P450s. Both GO enrichment and detection of genes showing similar expression patterns indicate that these other genes probably act together with the primary genes as part of the same metabolic detoxification pathway.

The upregulation of transposon-related genes in FUMOZ including the AFUN019794, (BEL12_AG transposon polyprotein; FC 83.2), is another feature of the differential expression between the two strains. We have previously reported that insertion of transposable elements is associated with the upregulation of detoxification genes in *An. funestus* (Weedall *et al.* 2020). Similar observations have been made in *Drosophila melanogaster* with the insertion the *Accord* transposable element into the 5′ end of the *CYP6G1* gene which drives its over-expression (Daborn *et al.* 2002). Transposable element insertions are commonly associated with disruption of gene function (Bourque *et al.* 2018), but in the case of *An. funestus*, they probably facilitate resistance by introducing additional cis-regulatory elements. Differential suppression of transposable elements could control the frequency at which insertions occur (Bourque *et al.* 2018). In most contexts, weaker suppression would be deleterious, and we speculate here for future exploration, that such an extreme strategy could reflect the strength of selection imposed by insecticides on mosquitoes.

Interestingly, a case of alternative splicing in the ABCA gene (AFUN019220) was strongly contrasted between strains suggesting a possible association with resistance as this gene has been shown to be over-expressed in pyrethroid-resistant populations Africa-wide (Weedall *et al.* 2019). Although this difference in isoform preference may also reflect the different geographic origins of the two strains, hence, further evidence is required to link this observation to insecticide resistance. A similar case of alternative splicing driving resistance to insecticide was reported in the tomato leaf miner, *Tuta absoluta* and shown to confer resistance to Spinosad insecticide through an exon skipping mechanism in the nicotinic acetylcholine receptor (nAChR) α6 subunit (Berger *et al.* 2016). The effect of differing alternative last exons (ALEs) in insects is poorly understood (Lee *et al.* 2021), however, in mice ALE choice directs subcellular localization of mRNAs (Taliaferro *et al.* 2016). Further work is needed to assess the association of the alternative splicing event at ABCA (AFUN019220) and pyrethroid resistance.

### Patterns of SNP polymorphism correlate with transcription profiles with reduced diversity in *rp1*

Analysis of the genetic diversity patterns between both strains revealed striking differences with marked reduced diversity in FUMOZ in genomic regions spanning major resistance loci notably *rp1* in the 2R chromosome. This result further supports the usefulness of RNAseq data to detect phenotypic signatures of selective sweep as the reduced diversity in FUMOZ using RNAseq correlates well with signatures previously detected with whole-genome sequencing in *An. funestus* (Barnes *et al.* 2017b; Weedall *et al.* 2020). The detection of the N384S mutation from *CYP6P9b* previously reported to drive an allelic variation conferring pyrethroid resistance to this species (Ibrahim *et al.* 2015) further shows the robustness of the frequency filtering approach used here in detecting phenotypically meaningful variants from RNAseq data. Analysis of the polymorphism patterns of major resistance genes *CYP6P9a* and *CYP6P9b* confirms that the reduced diversity in FUMOZ is likely associated with pyrethroid resistance since other genes such as *CYP4C36* did not show the same difference in polymorphisms. Similar correlation between patterns of genetic diversity from RNAseq and genome sequencing has been reported recently in the almond agroecosystem (Calla *et al.* 2021).

One of the significant observations was the correlation between the reduced diversity around *rp1* and the high over-expression of *CYP6P9a and CYP6P9b* supporting that metabolic resistance in *An. funestus* operates through both over-expression of detoxification genes but also through the selection of a highly metabolically active allele in line with the role of allelic variation shown previously for the duplicated *CYP6P9a/b* genes (Ibrahim *et al.* 2015). Similar correlation between over-expression and strong signatures of selective sweep have been reported before in *An. funestus* for other detoxification genes including GSTe2 (Riveron *et al.* 2014; Weedall *et al.* 2020), for *CYP9K1* (Weedall *et al.* 2020), CYP6 cluster on 2R chromosome in *An. gambiae* as revealed by the 1000Ag genomes study (Anopheles gambiae 1000 Genomes Consortium 2017), for the *CYP6G1* in *Drosophila* (Schlenke and Begun 2004) or in the almond agroecosystem (Calla *et al.* 2021).

Not all significant SNPs in detoxification genes correlate with differential expression. For example, the *CYP6Z1*, *CYP6M7*, *CYP6Z3* genes of the resistance to pyrethroid 2 (*rp2*) locus all previously shown to confer pyrethroid resistance (Irving *et al.* 2012; Ibrahim *et al.* 2016). These genes are particularly intriguing as the *rp2* locus has not been predominant in resistant in natural populations across Africa, including samples from southern Africa sampled more recently than the FUMOZ strain (Weedall *et al.* 2019). Neither were the *CYP4G17* and *CYP4G16* P450 genes shown to be associated with cuticular resistance in *An. gambiae* (Balabanidou *et al.* 2016). Perhaps the most important role for these P450s is in the larval and pupal stage and we may have missed the peak of expression for these genes in our adult samples. Alternatively, as their expression is restricted to cuticular hydrocarbon secreting oenocytes (Balabanidou *et al.* 2016) any resistance-associated signal was swamped by our whole organism approach. As cuticular resistance is thought likely to confer cross-resistance by creating a barrier to insecticide permeability (Balabanidou *et al.* 2018), the variants identified here warrant further investigation.

## Conclusion

The comparative analysis of the transcription profiles of two phenotypically contrasted laboratory strains of the malaria vectors *An. funestus* has highlighted specific features associated with pyrethroid resistance. These include the predominance of cytochrome P450 genes in association with previous QTL studies; the strong association between over-expression and reduced diversity of major resistance genes; a potential role of alternative splicing from ABC transporter and the co-expression of several genes belonging to different families to confer resistance to mosquitoes. The detection of SNPs of interest and signatures of selective sweep and using RNAseq have also been achieved supporting the usefulness of this method beyond transcription profiling. This study will further facilitate future studies on the molecular basis of resistance in field populations of mosquitoes.

## Data availability

RNAseq data submitted to the European Nucleotide Archive under PRJEB45224.

Supplementary material is available at *G3* online.

## Supplementary Material

jkab352_Supplementary_Data
