# Supplementary material for: RNAseq-based gene expression profiling of the Anopheles funestus pyrethroid-resistant strain FUMOZ highlights the predominant role of the duplicated CYP6P9a/b cytochrome P450s
Source: G3 (Bethesda). 2021 Oct 9;12(1):jkab352. doi: 10.1093/g3journal/jkab352 (PMC8727960; doi:10.1093/g3journal/jkab352)
Supplement: jkab352_Supplementary_Data [file jkab352_supplementary_data.pdf]

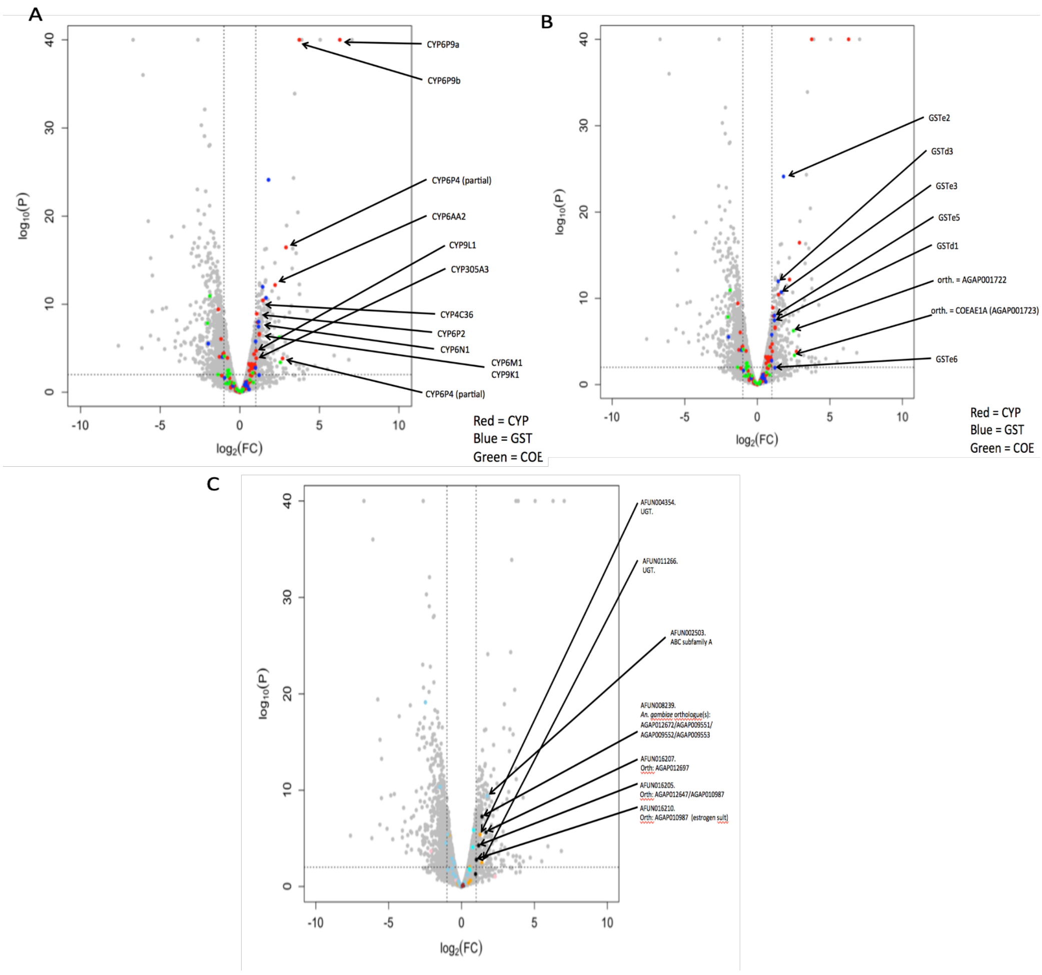

Figure S1: Volcano plot of differential expression of major detoxification gene families between FUMOZ and FANG: A) Main over-expressed cytochrome P450 genes in FUMOZ (in red); C) Main over-expressed glutathione S-transferase genes in FUMOZ (in blue); C) Other over-expressed detoxification gene families in FUMOZ (in red).

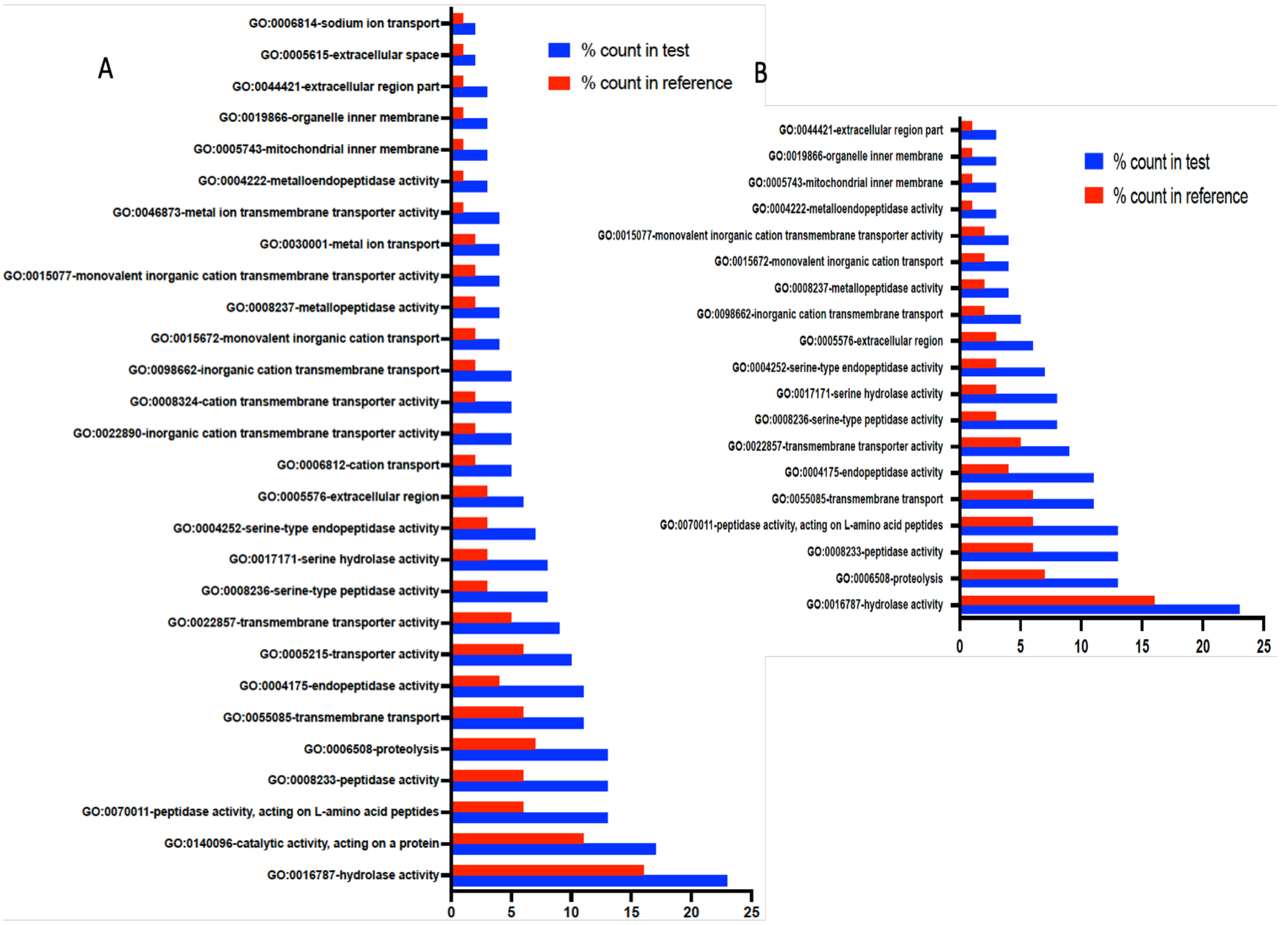

Figure S2: Gene Ontology (GO) terms enriched in transcripts up-regulated (A) and downregulated (B) in FUMOZ using BLAST2GO. Significance of the enrichment was assessed using a Fisher's exact test and Benjamini and Hochberg multiple testing correction (P< 0.05).

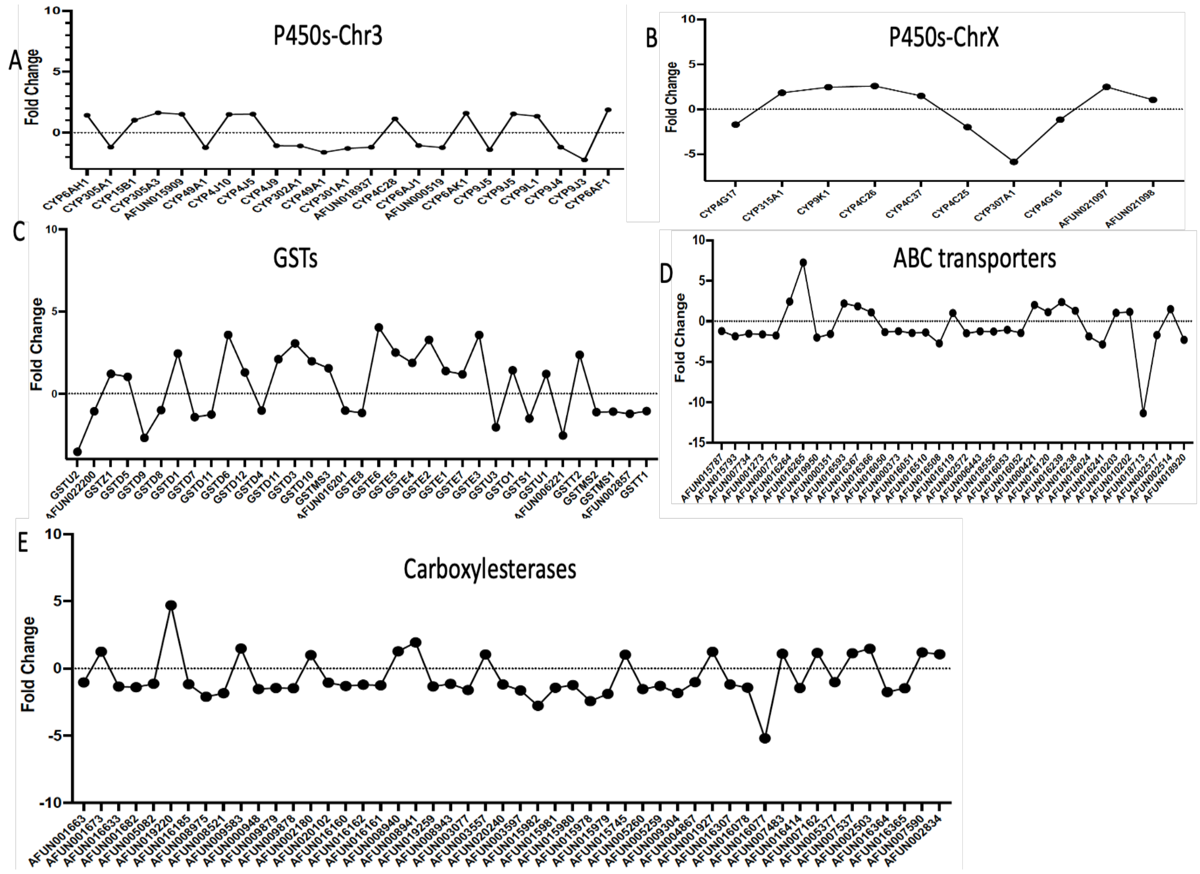

Figure S3: Global expression profile of major detoxification gene families across the genome: A) Gene expression pattern of all cytochrome P450 genes on chromosome 3. Value above 0 indicates over-expression in FUMOZ, whereas below 0 is down-regulation compared to FANG. B) is for Cytochrome P450s on X chromosomes. C) Gene Expression patterns of all GSTs. D) Gene expression patterns of all ABC transporter genes across the genome. E) Gene expression patterns of all carboxylesterase genes across the genome.

Table S1: List of qRT-PCR primers

Gene	Forward primer	Reverse primer	Expected size (bp)
		**Primers used for qRT-PCR**	
**CYP6P9a**	CAGCGCGTACACCAGATTGTGTAA	TCACAATTTTTCCACCTTCAAGTAATTACCCGC	92
**CYP6P9b**	CAGCGCGTACACCAGATTGTGTAA	TTACACCTTTTCTACCTTCAAGTAATTACCCGC	97
**CYP6AA1**	CATCTGGCTGAATGGCACTA	TCAACAATGCCATCAAATCG	109
**GSTe2**	GTTTGAAGCAGTTGCCATACTACGAGG	TCAAGCTTTAGCATTTTCCTCCTTTTTGGC	101
**CYP6P5**	ACGTAATCAACGAAACGCTTCGTA	TCGGTATCTGCACGATTGTT	126
**CYP325A**	GGATACCGATACGGCATGTT	TGCGTATTTGATGCCCTACA	148
**RSP7**	GTGTTCGGTTCCAAGGTGAT	TCCGAGTTCATTTCCAGCTC	98
**ACTIN**	TTAAACCCAAAAGCCAATCG	ACCGGATGCATACAGTGACA	111

Tables S2: Alignment statistics

	FANG1	FANG2	FANG3	FANG4	FUMOZ1	FUMOZ2	FUMOZ3	FUMOZ4
**Normal**	28532364 (47.35)	35604808 (81.85)	35809448 (65.72)	47770060 (79.32)	46362088 (83.46)	46921040 (82.42)	47546860 (82.59)	54134584 (82.26)
**One mate flip**	131108 (0.22)	127896 (0.29)	159186 (0.29)	151668 (0.25)	167300 (0.3)	210360 (0.37)	174518 (0.3)	191224 (0.29)
**Both mate flip**	448096 (0.74)	611212 (1.41)	606734 (1.11)	793194 (1.32)	760376 (1.37)	703322 (1.24)	757380 (1.32)	887758 (1.35)
**Translocated**	84316 (0.14)	83372 (0.19)	87116 (0.16)	59554 (0.1)	72126 (0.13)	117382 (0.21)	104058 (0.18)	95094 (0.14)
**Mate missing**	1632069 (2.71)	1007629 (2.32)	1824059 (3.35)	1220564 (2.03)	963095 (1.73)	961597 (1.69)	1050907 (1.83)	1219629 (1.85)
**Unaligned**	29436108 (48.85)	6067197 (13.95)	16001557 (29.37)	10231588 (16.99)	7225042 (13.01)	8014273 (14.08)	7937523 (13.79)	9280205 (14.1)
**chr3**	13805642 (44.78)	15550846 (41.54)	17103511 (44.44)	21310072 (42.62)	20451495 (42.32)	20239650 (41.38)	20626024 (41.56)	23831609 (42.16)
**chr2**	14652708 (47.53)	18898960 (50.48)	18371638 (47.74)	24608550 (49.22)	24133412 (49.94)	24915200 (50.94)	25235854 (50.84)	28450662 (50.33)
**chrX**	2369530 (7.69)	2985002 (7.97)	3011355 (7.82)	4076393 (8.15)	3740060 (7.74)	3758827 (7.68)	3771821 (7.6)	4245996 (7.51)
**Total number of reads**	60264061	43502114	54488100	60226628	55550027	56927974	57571246	65808494

Table S3: Top 20 over-expressed genes in FUMOZ compared to FANG

Gene ID	Q value	FC	Log FC	FANG	FUMOZ	Description	Chr
AFUN019794	3.79E-06	90.47	6.50	520.44	67882.05		chr2
AFUN014490	0.0013	82.23	6.36	5.26	410.85		chr3
AFUN015792	1.08E-05	71.49	6.16	822.61	84176.54	cytochrome P450; CYP6P9a	chr2
AFUN007421	0.00179	68.99	6.11	6.18	428.24		chr2
AFUN019207	3.70E-06	68.98	6.11	271.30	27474.14		chr2
AFUN012016	8.68E-08	66.49	6.06	0.25	81.39		chr3
AFUN018641	0.001703	31.03	4.96	0.98	64.15	Ficolin-1;	chr3
AFUN019813	8.36E-04	24.92	4.64	121.40	4612.43	Retrovirus-related Pol polyprotein from transposon TNT 1-94	chrX
AFUN010785	1.45E-04	21.74	4.44	313.78	9454.57	Chromodomain-helicase-DNA-binding protein 4;	chr3
AFUN018981	0.014188	19.93	4.32	19.48	306.09	Transmembrane protease serine 11D; AltName:	chr3
AFUN020923	0.00617	19.85	4.31	3.11	57.63	cuticular protein RR-2 family	chr2
AFUN019546	4.64E-04	18.68	4.22	12.98	350.83	Histone acetyltransferase Tip60	chr2
AFUN020946	0.008556	16.36	4.03	10.77	153.93	serine-type enodpeptidase; chymotrypsin-2	chr3
AFUN021228	0.001358	16.28	4.02	8.99	227.10	Golgi SNAP receptor complex member	chr2
AFUN004822	9.68E-04	15.30	3.94	19.66	405.09		chr3
AFUN021240	3.70E-04	15.06	3.91	46.18	1050.18	Carboxypeptidase Q; AltName:	chr2
AFUN005683	1.45E-04	14.84	3.89	6.36	130.78	THAP domain-containing protein 6	chrX
AFUN011284	0.017727	12.28	3.62	13.68	149.19		chr2
AFUN015252	2.01E-04	11.73	3.55	0.15	14.87		chr3
AFUN015889	6.90E-05	11.15	3.48	744.25	11906.60	cytochrome P450: CYP6P9b	chr2
AFUN019850	0.016703	10.87	3.44	5.36	46.00		chr3
AFUN009695	1.58E-04	10.84	3.44	0.14	13.83		chr3
AFUN017249	0.01925	10.08	3.33	11.50	99.50	5S ribosomal RNA	chr3

Table S4: Down-regulated transcripts in FUMOZ compared to FANG

Gene ID	Corrected P value	FC	Log FC	Gene Symbol	FUMOZ	FANG	Description	Chrom
				**Most downregulated in FUMOZ-R**				
AFUN001823	0.0002	-602.4	-9.2	AFUN001823	12.8	581.9	Group XIIB secretory phospholipase A2-like protein	chr3
AFUN019775	0.0054	-548.1	-9.1	AFUN019775	9.3	4063.4	Heat shock protein 70 A1	chr2
AFUN019570	0.0051	-324.6	-8.3	AFUN019570	45.1	12679.1	Heat shock protein 70 B2	chr2
AFUN003205	0.0002	-283.3	-8.1	AFUN003205	0.7	227.0	unknown	chr3
AFUN019671	0.0067	-236.9	-7.9	AFUN019671	37.3	7994.0	Heat shock protein 70 B2	chr2
AFUN019513	0.0067	-206.3	-7.7	AFUN019513	27.9	4514.6	Heat shock protein 70 B2	chr2
AFUN018693	0.0005	-202.4	-7.7	AFUN018693	10.9	1313.3	Thioredoxin peroxidase 4	chr3
AFUN019178	0.0064	-151.2	-7.2	AFUN019178	20.2	2924.8	Heat shock protein 70 B2	chr2
AFUN017562	0.0310	-148.9	-7.2	SSU_rRNA	36.4	4445.2	Eukaryotic small subunit ribosomal RNA	chr3
AFUN019467	0.0082	-147.0	-7.2	AFUN019467	59.0	8423.1	Heat shock protein 70 B2	chr2
AFUN022250	0.0144	-144.4	-7.2	AFUN022250	34.6	1446.5	Serine proteinase stubble;	chr3
AFUN001824	0.0001	-139.3	-7.1	AFUN001824	31.0	644.8	C2 domain-containing protein	chr3
AFUN019289	0.0069	-134.0	-7.1	AFUN019289	34.7	3839.6	Heat shock protein 70 B2	chr2
AFUN006337	0.0006	-130.2	-7.0	AFUN006337	13.8	805.6	chitinase	chr2
AFUN017680	0.0233	-114.7	-6.8	LSU_rRNA	29.8	1026.5	Eukaryotic large subunit ribosomal RNA	chr3
AFUN017729	0.0125	-85.5	-6.4	LSU_rRNA	2.5	190.3	Eukaryotic large subunit ribosomal RNA	chr3
AFUN017400	0.0367	-79.8	-6.3	LSU_rRNA	3.3	298.7	Eukaryotic large subunit ribosomal RNA	chr3
AFUN017608	0.0403	-72.8	-6.2	LSU_rRN	2.1	242.2	Eukaryotic large subunit ribosomal RNA	chr3
AFUN018757	0.0005	-62.9	-6.0	AFUN018757	2.5	94.4	Small integral membrane protein 20	chr3
				**Cytochrome P450s**				
AFUN020636	0.0096	-2.4	-1.3	AFUN020636	132.1	238.7	cytochrome P450	chr2
AFUN020179	0.0044	-2.4	-1.2	AFUN020179	255.7	386.4	cytochrome P450	chr2
AFUN015714	0.0161	-2.1	-1.1	CYP6AD1	35.4	40.4	cytochrome P450	chr2
AFUN015866	0.0119	-2.2	-1.2	CYP9J3	1371.5	2206.3	cytochrome P450	chr3
AFUN006122	0.0043	-2.8	-1.5	CYP4C27	514.9	950.1	cytochrome P450	chr2
				**ABC transporters**				
AFUN016077	0.0045	-5.2	-2.4	AFUN016077	3777.8	14533.4	ATP-binding cassette transporter A	chr3
AFUN015978	0.0051	-2.4	-1.3	AFUN015978	262.5	387.8	ATP-binding cassette transporter G	chr2
AFUN015982	0.0104	-2.8	-1.5	AFUN015982	1171.8	2293.4	ATP-binding cassette transporter G	chr2
AFUN008975	0.0042	-2.1	-1.1	AFUN008975	4980.4	6779.1	ATP-binding cassette transporter H	chr2
				**Carboxylesterases**				
AFUN019950	0.0123	-2.0	-1.0	AFUN019950	806.5	1007.2	carboxylesterase	chr2
AFUN016508	0.0042	-2.7	-1.5	AFUN016508	39.7	55.0	carboxylesterase juvenile hormone esterase	chr3
AFUN018713	0.0051	-11.4	-3.5	AFUN018713	66.4	474.7	Carboxylic ester hydrolase	chr3
AFUN016241	0.0491	-2.9	-1.5	AFUN016241	267.5	426.8	Carboxylic ester hydrolase	chr3
				**Others**				
AFUN003809	0.0222	-2.1	-1.1	CSP3	111.1	147.9	chemosensory protein 3	chr2
AFUN008656	0.0101	-55.8	-5.8	AFUN008656	131.5	4350.1	alpha-crystallin B chain	chr3
AFUN001821	0.0018	-3.3	-1.7	GRX1	177.1	369.3	glutaredoxin	chr3

AFUN006221	0.0018	-2.5	-1.4	AFUN006221	310.1	486.7	Glutathione peroxidase	chrX
AFUN008426	0.0087	-3.5	-1.8	GSTU2	60.9	159.1	glutathione S-transferase	chr2

Table S5: Differentially alternatively spliced genes between FUMOZ and FANG

Gene ID	Splicing Index	Transcript	FANG	FUMOZ	Description	Chrom
AFUN021186	0.77	AFUN021186-RA	192.4	67.0	DEAD-box ATP-dependent RNA helicase 30	chr3
AFUN019220	0.75	AFUN019220-RB	654.1	4429.5	ATP-binding cassette sub-family A member 3	chr2
AFUN020353	0.60	AFUN020353-RA	1766.2	1355.0	6-phosphofructo-2-kinase/fructose-2,6-bisphosphatase	chr3
AFUN002009	0.58	AFUN002009-RB	33589.5	50074.6	60S ribosomal protein L4	chr2
AFUN004135	0.49	AFUN004135-RB	2938.1	5141.9	Nucleosomal histone kinase 1	chr3
AFUN011313	0.42	AFUN011313-RA	100.8	252.7	Dihydropyrimidinase	chr2
AFUN011575	0.42	AFUN011575-RA	162.3	221.7	Paired box protein Pax-6	chrX
AFUN020206	0.36	AFUN020206-RA	23899.1	29997.9	Protein kinase C, brain isozyme	chr3
AFUN007971	0.35	AFUN007971-RA	29469.0	15671.5	Tropomyosin-2; AltName	chr2
AFUN021345	0.35	AFUN021345-RA	371.8	578.8	Protein FAM151B	chr2
AFUN011621	0.35	AFUN011621-RA	3958.3	3145.5	Papilin; Flags: Precursor	chr2
AFUN020059	0.34	AFUN020059-RA	38.1	52.3	Serine proteinase stubble; AltName	chr2
AFUN020405	0.34	Novel	7193.7	6702.0	Sodium/calcium exchanger 3; AltName	chr2
AFUN016021	0.34	AFUN016021-RB	52162.3	64984.9	Fructose-bisphosphate aldolase	chr2
AFUN000373	0.33	AFUN000373-RA	375.8	488.5	Esterase E4; carboxylesterase	chr3
AFUN008882	0.31	AFUN008882-RA	3828.9	4649.0	Mitochondrial uncoupling protein 4	chr2
AFUN007786	0.31	AFUN007786-RB	3463.0	2859.9	Cytosolic carboxypeptidase-like protein 5	chr3
AFUN001285	0.30	AFUN001285-RA	2371.0	2974.0	Uncharacterized family 31 glucosidase KIAA1161	chr2
AFUN001732	0.29	AFUN001732-RA	1671.6	2565.7	Putative transmembrane protein INAFM2	chrX
AFUN001485	0.27	AFUN001485-RB	4791.7	5655.5	Dedicator of cytokinesis protein 2	chr2
AFUN011127	0.26	AFUN011127-RA	317.2	329.2	Low-density lipoprotein receptor-related protein 6	chr3
AFUN003820	0.26	AFUN003820-RB	974.1	422.4	Nicotinate phosphoribosyltransferase	chr2
AFUN019842	0.26	AFUN019842-RB	20074.7	25807.7	Ankyrin repeat and KH domain-containing protein mask;	chr2
AFUN011633	0.26	Novel	2753.0	3021.4	Glutathione-specific gamma-glutamylcyclotransferase 1	chr2
AFUN020766	0.25	AFUN020766-RB	8754.0	10578.1	Serine/threonine-protein kinase Genghis Khan	chr3
AFUN020610	0.25	AFUN020610-RA	597.2	920.7	Serine palmitoyltransferase small subunit A	chr2
AFUN010515	0.25	AFUN010515-RA	1677.9	1429.3	Tubulin-specific chaperone cofactor E-like protein	chr3

Table S6: Similarity of expression to key entities

Gene ID	Similarity	FANG	FUMOZ	Gene Symbol	Description	Chrom
				**CYP6P9a**		
AFUN015792	1.00	795.2	83547.3	CYP6P9a	Cytochrome P450	chr2
AFUN012016	0.92	1.0	80.9	AFUN012016		chr3
AFUN019207	0.89	267.9	27164.2	AFUN019207		chr2
AFUN019794	0.88	506.0	67281.0	AFUN019794		chr2
AFUN010785	0.77	295.4	9436.6	AFUN010785	Chromodomain-helicase-DNA-binding protein 4	chr3
AFUN019813	0.73	113.7	4163.2	AFUN019813	Retrovirus-related Pol polyprotein from transposon TNT 1-94	chrX
AFUN019546	0.73	12.4	340.2	AFUN019546	Histone acetyltransferase Tip60	chr2
AFUN018641	0.72	3.9	52.7	AFUN018641	Ficolin-1; AltName: Full	chr3
AFUN005683	0.69	6.0	130.4	AFUN005683	THAP domain-containing protein 6	chrX
AFUN021240	0.67	45.2	1000.2	AFUN021240	Carboxypeptidase Q	chr2
AFUN021228	0.66	8.3	199.4	AFUN021228	Golgi SNAP receptor complex member 1	chr2
AFUN015889	0.63	723.4	11853.3	CYP6P9b	Cytochrome P450	chr2
				**CYP6P9b**		
AFUN015889	1.00	723.4	11853.3	CYP6P9b	cytochrome P450	chr2
AFUN019220	0.77	654.1	4429.5	ABCA	ATP-binding cassette sub-family A member 3	chr2
AFUN020895	0.76	242.9	1825.3	CYP6P4a	Cytochrome P450	chr2
AFUN000622	0.74	220.4	1303.9	AFUN000622	Solute carrier family 23 member 2	chr2
AFUN019813	0.73	113.7	4163.2	AFUN019813	Retrovirus-related Pol polyprotein from transposon TNT 1-94	chrX
AFUN021239	0.73	63.3	758.3	AFUN021239	Carboxypeptidase Q	chr2
AFUN022201	0.72	4.3	48.6	AFUN022201	Glutathione peroxidase	chr2
AFUN015936	0.72	107.7	713.7	Gr20	Gustatory receptor for sugar taste 64a	chr2
AFUN016008	0.71	112.7	595.5	GSTE6	Glutathione S-transferase 1	chr2
AFUN016265	0.71	43.9	370.6	AFUN016265	carboxylesterase	chr2
AFUN015809	0.71	486.9	2392.7	GSTE2	Glutathione S-transferase	chr2
				**GSTe2**		
AFUN015809	1.00	486.9	2392.7	GSTE2	Glutathione S-transferase	chr2
AFUN020670	0.91	242.8	983.8	AFUN020670	Xanthine dehydrogenase	chr3
AFUN006135	0.90	249.3	972.0	CYP4C36	Cytochrome P450	chr2
AFUN015811	0.89	329.2	1174.8	GSTE5	Glutathione S-transferase	chr2
AFUN007549	0.89	1041.9	3930.9	CYP9K1	cytochrome P450	chrX
AFUN015839	0.88	319.2	1293.9	GSTD3	Glutathione S-transferase	chr2
AFUN008239	0.86	217.2	818.6	AFUN008239	Sulfotransferase family cytosolic 1B member 1	chr2
AFUN004002	0.86	4595.5	16720.6	AFUN004002	Arginosuccinate lyase	chr3
AFUN010918	0.86	385.0	1182.4	CYP6N1	cytochrome P450	chr2
AFUN015801	0.86	702.7	2100.6	CYP6P2	cytochrome P450	chr2
AFUN016010	0.85	4085.5	12921.1	GSTD1	Glutathione S-transferase	chr2
AFUN011515	0.85	1178.3	3512.7	TPX4	Peroxiredoxin-6	chr3
AFUN016264	0.85	70.7	232.4	AFUN016264	Carboxylesterase	chr2
AFUN016210	0.85	89.0	288.0	AFUN016210	Sulfotransferase family cytosolic 1B member 1	chr3

AFUN016008	0.84	112.7	595.5	GSTE6	Glutathione S-transferase	chr2
AFUN015808	0.83	738.1	3158.6	GSTE3	Glutathione S-transferase	chr2
AFUN015938	0.83	575.8	1872.9	CYP9M1	cytochrome P450	chr2
AFUN016207	0.81	140.2	666.5	AFUN016207	Sulfotransferase family cytosolic 1B member 1	chr3
AFUN020895	0.81	242.9	1825.3	AFUN020895	cytochrome P450	chr2
AFUN015767	0.80	20.3	63.5	GSTD11	Glutathione S-transferase	chr2
AFUN011266	0.77	55.6	168.0	AFUN011266	UDP-glucuronosyltransferase 3A1	chr2
				**ABCA-AFUN019220**		
AFUN019220	1.00	654.1	4429.5	AFUN019220	ATP-binding cassette sub-family A member 3	chr2
AFUN015809	0.89	486.9	2392.7	GSTE2	Glutathione S-transferase	chr2
AFUN016008	0.86	112.7	595.5	GSTE6	Glutathione S-transferase	chr2
AFUN006135	0.84	249.3	972.0	CYP4C36	Cytochrome P450	chr2
AFUN015839	0.84	319.2	1293.9	GSTD3	Glutathione S-transferase	chr2
AFUN020670	0.83	242.8	983.8	AFUN020670	Xanthine dehydrogenase	chr3
AFUN020895	0.83	242.9	1825.3	AFUN020895	Cytochrome P450	chr2
AFUN016207	0.82	140.2	666.5	AFUN016207	Sulfotransferase family cytosolic 1B member 1	chr3
AFUN008239	0.81	217.2	818.6	AFUN008239	Sulfotransferase family cytosolic 1B member 1	chr2
AFUN015811	0.81	329.2	1174.8	GSTE5	Glutathione S-transferase	chr2
AFUN007549	0.80	1041.9	3930.9	CYP9K1	Cytochrome P450	chrX
AFUN016205	0.79	354.1	1253.1	AFUN016205	Sulfotransferase family cytosolic 1B member 1	chr3
AFUN015808	0.79	738.1	3158.6	GSTE3	Glutathione S-transferase	chr2
AFUN019602	0.78	377.2	1376.1	AFUN019602	Solute carrier family 46 member 3	chr2
AFUN015785	0.78	247.5	775.4	CYP6AA2	Cytochrome P450	chr2
AFUN015801	0.78	702.7	2100.6	CYP6P2	Cytochrome P450	chr2
AFUN010918	0.78	385.0	1182.4	CYP6N1	Cytochrome P450	chr2
AFUN016010	0.78	4085.5	12921.1	GSTD1	Glutathione S-transferase	chr2
AFUN004002	0.77	4595.5	16720.6	AFUN004002	Argininosuccinate lyase; Short	chr3
AFUN016264	0.77	70.7	232.4	AFUN016264	Carboxylesterase	chr2
AFUN015767	0.77	20.3	63.5	GSTD11	Glutathione S-transferase	chr2
AFUN016210	0.76	89.0	288.0	AFUN016210	Sulfotransferase family cytosolic 1B member 1	chr3
AFUN011515	0.76	1178.3	3512.7	TPX4	Thioredoxin peroxidase 4	chr3

Table S7: Genes with significant SNPs between FUMOZ and FANG with contrasting percentage of supporting reads of 90-100% in FANG and 0-10% in FUMOZ in comparison to the reference AFUN3.1 genome from FUMOZ strain

Transcript	Chrom	Start	Non Syn	Syn	Int	5'	3'	Gene	Description
AFUN015786	chr2	8531151	0	7	0	0	1	CYP6AA1	Cytochrome P450
AFUN015785	chr2	8533523	1	0	0	0	3	CYP6AA2	Cytochrome P450
AFUN015792	chr2	8544505	0	6	0	0	0	CYP6P9A	Cytochrome P450
AFUN015889	chr2	8554228	2	2	0	0	2	CYP6P9b	Cytochrome P450
AFUN020895	chr2	8559923	0	1	0	0	0	CYP6P4a	Cytochrome P450
AFUN015802	chr2	8565228	0	1	0	0	0	CYP6P1	Cytochrome P450
AFUN015801	chr2	8567086	1	6	0	0	1	CYP6P2	Cytochrome P450
AFUN019220	chr2	12554528	2	0	0	0	0	AFUN019220	ATP-binding cassette transporter (ABCA)
AFUN005864	chr2	16472020	0	1	0	0	1	CYP4AR1	Cytochrome P450
AFUN015992	chr2	16479851	1	0	0	0	0	CYP4D17	Cytochrome P450
AFUN015790	chr2	18835030	0	1	0	0	0	CYP6AG1	Cytochrome P450
AFUN015791	chr2	18838665	0	4	2	0	2	CYP6AG2	Cytochrome P450
AFUN008975	chr2	29275431	0	11	3	2	0	AFUN008975	ATP-binding cassette transporter (ABCH)
AFUN008521	chr2	31863331	0	2	2	0	2	AFUN008521	ATP-binding cassette transporter (ABCH)
AFUN002180	chr2	37885682	0	4	0	1	1	AFUN002180	ATP-binding cassette
AFUN016010	chr2	41556837	0	0	92	0	0	GSTD1	glutathione S-transferase
AFUN010604	chr2	63336975	1	1	0	0	0	HPX2	heme peroxidase 2
AFUN008941	chr2	72303465	0	1	0	0	0	AFUN008941	ATP-binding cassette transporter (ABCC)
AFUN003077	chr2	77004788	0	1	1	0	0	AFUN003077	ATP-binding cassette transporter (ABCG)
AFUN003557	chr2	77281890	0	0	70	0	0	AFUN003557	ATP-binding cassette transporter (ABCC)
AFUN005623	chr2	77431433	0	0	8	0	36	AFUN005623	CCR4-NOT transcription complex subunit
AFUN020240	chr2	77624199	0	1	0	0	0	AFUN020240	ATP-binding cassette transporter (ABCC)
AFUN000819	chr2	79102501	0	5	0	0	1	GRX3	glutaredoxin
AFUN015918	chr2	82885114	1	6	0	3	0	CYP6Z3	Cytochrome P450
AFUN015919	chr2	82887380	1	5	0	1	1	CYP6Z1	cytochrome P450
AFUN015795	chr2	85291221	0	0	0	1	0	CYP6M7	Cytochrome P450
AFUN015794	chr2	85294344	1	1	0	0	0	CYP6M2	Cytochrome P450
AFUN010918	chr2	85300817	0	3	0	0	0	CYP6N1	Cytochrome P450
AFUN020636	chr2	85303624	0	0	0	0	2	AFUN020636	Cytochrome P450
AFUN015962	chr2	85315445	1	3	0	3	1	CYP6N2	Cytochrome P450

AFUN015775	chr2	87479299	0	0	0	1	0	CYP12F2	Cytochrome P450
AFUN015774	chr2	87484861	1	1	0	0	0	CYP12F4	Cytochrome P450
AFUN003597	chr2	90831993	0	1	0	0	0	AFUN003597	ATP-binding cassette
AFUN015982	chr2	94143669	0	0	0	0	1	AFUN015982	ATP-binding cassette transporter (ABCG)
AFUN015980	chr2	94152192	1	2	0	0	0	AFUN015980	ATP-binding cassette transporter (ABCG)
AFUN015745	chr2	94162941	0	2	1	1	0	AFUN015745	ATP-binding cassette transporter (ABCG)
AFUN005260	chr2	94177266	2	8	21	1	2	AFUN005260	ATP-binding cassette transporter (ABCG)
AFUN005259	chr2	94200172	1	6	5	0	1	AFUN005259	ATP-binding cassette transporter (ABCG)
AFUN020179	chr2	96631587	1	0	0	0	0	AFUN020179	Cytochrome P450
AFUN006122	chr2	96816472	0	1	0	0	0	CYP4C27	Cytochrome P450
AFUN004867	chr3	8719813	0	2	0	0	3	AFUN004867	ATP-binding cassette
AFUN015907	chr3	8957122	0	1	0	0	0	CYP305A3	Cytochrome P450
AFUN016050	chr3	10052763	0	2	0	0	0	AFUN016050	Carboxylic ester hydrolase
AFUN015904	chr3	12808612	1	0	0	0	0	CYP4J5	Cytochrome P450
AFUN007483	chr3	32260692	0	1	0	0	0	AFUN007483	ATP-binding cassette
AFUN008829	chr3	56790189	0	0	4	0	0	GSTS1	Glutathione S-transferase
AFUN016414	chr3	57053136	0	1	0	0	0	AFUN016414	ATP-binding cassette transporter (ABCA)
AFUN007162	chr3	61776505	3	21	11	4	2	AFUN007162	ATP-binding cassette transporter (ABCE)
AFUN016024	chr3	65265250	0	1	0	0	1	AFUN016024	carboxylesterase
AFUN010202	chr3	70745052	0	1	0	0	0	AFUN010202	carboxylesterase
AFUN001382	chr3	73206528	1	7	0	0	2	CYP9J5	Cytochrome P450
AFUN001383	chr3	73210543	0	1	0	0	0	CYP9J5	Cytochrome P450
AFUN015862	chr3	73267772	1	5	0	0	0	CYP9L1	Cytochrome P450
AFUN015865	chr3	73273647	2	8	0	0	1	CYP9J4	Cytochrome P450
AFUN015866	chr3	73276616	3	8	0	0	0	CYP9J3	Cytochrome P450
AFUN005377	chr3	73746552	1	6	0	0	0	AFUN005377	ATP-binding cassette
AFUN009234	chr3	77067934	0	2	0	0	0	GSTU1	Glutathione S-transferase
AFUN007537	chr3	82705450	0	0	0	0	1	AFUN007537	ATP-binding cassette
AFUN002517	chr3	86926810	0	1	0	0	0	AFUN002517	carboxylesterase
AFUN011481	chrX	2974301	0	2	0	0	0	CYP4G17	Cytochrome P450
AFUN007590	chrX	7980432	0	1	0	4	1	AFUN007590	ATP-binding cassette
AFUN002155	chrX	10690535	0	0	2	0	0	GSTMS1	Glutathione transferase microsomal
AFUN010428	chrX	16589547	0	3	4	2	0	CYP4G16	Cytochrome P450

Table S8: Coding region SNPs in Cytochrome P450s between FUMOZ and FANG with contrasting percentage of supporting reads of 90-100% in FANG and 0-10% in FUMOZ in comparison to the reference AFUN3.1 genome from FUMOZ strain

Transcripts	Gene Name	Nucleotide	Amino acid	Start	Chrom	Type of SNPs
AFUN015786	CYP6AA1	951T>G	Thr317Thr	8532239	chr2	Synonymous
AFUN015786	CYP6AA1	936C>T	Phe312Phe	8532254	chr2	Synonymous
AFUN015786	CYP6AA1	891T>G	Leu297Leu	8532299	chr2	Synonymous
AFUN015786	CYP6AA1	858T>C	Asp286Asp	8532332	chr2	Synonymous
AFUN015786	CYP6AA1	543C>T	Cys181Cys	8532647	chr2	Synonymous
AFUN015786	CYP6AA1	252G>A	Leu84Leu	8532938	chr2	Synonymous
AFUN015786	CYP6AA1	250T>C	Leu84Leu	8532940	chr2	Synonymous
AFUN015785	CYP6AA2	291T>A	His97Gln	8535206	chr2	NON_Synonymous
AFUN015792	CYP6P9A	753A>G	Leu251Leu	8545397	chr2	Synonymous
AFUN015792	CYP6P9A	654G>A	Leu218Leu	8545496	chr2	Synonymous
AFUN015792	CYP6P9A	457C>T	Leu153Leu	8545693	chr2	Synonymous
AFUN015792	CYP6P9A	189G>T	Leu63Leu	8545961	chr2	Synonymous
AFUN015792	CYP6P9A	186_187 delinsGT	Glu62_Leu63 delinsGluLeu	8545962	chr2	Synonymous
AFUN015889	CYP6P9b	1151A>G	Asn384Ser	8554721	chr2	NON_Synonymous
AFUN015889	CYP6P9b	723A>C	Ile241Ile	8555206	chr2	Synonymous
AFUN015889	CYP6P9b	457C>T	Leu153Leu	8555472	chr2	Synonymous
AFUN015889	CYP6P9b	307A>G	Ile103Val	8555622	chr2	NON_Synonymous
AFUN015802	CYP6P1	183A>G	Gln61Gln	8566692	chr2	Synonymous
AFUN015801	CYP6P2	1428C>A	Arg476Arg	8567509	chr2	Synonymous
AFUN015801	CYP6P2	963G>C	Thr321Thr	8568047	chr2	Synonymous
AFUN015801	CYP6P2	861A>G	Gln287Gln	8568149	chr2	Synonymous
AFUN015801	CYP6P2	850T>C	Leu284Leu	8568160	chr2	Synonymous
AFUN015801	CYP6P2	843A>G	Thr281Thr	8568167	chr2	Synonymous
AFUN015801	CYP6P2	701T>C	Leu234Ser	8568309	chr2	NON_Synonymous
AFUN015801	CYP6P2	372A>G	Ala124Ala	8568638	chr2	Synonymous
AFUN005864	CYP4AR1	891T>C	Asn297Asn	16472863	chr2	Synonymous
AFUN015992	CYP4D17	801C>G	Asp267Glu	16481490	chr2	NON_Synonymous
AFUN015790	CYP6AG1	135T>C	Ser45Ser	18835284	chr2	Synonymous
AFUN015791	CYP6AG2	276C>G	Val92Val	18840608	chr2	Synonymous
AFUN015791	CYP6AG2	279A>G	Leu93Leu	18840611	chr2	Synonymous
AFUN015791	CYP6AG2	393A>T	Thr131Thr	18840725	chr2	Synonymous
AFUN015791	CYP6AG2	1341G>A	Ser447Ser	18841902	chr2	Synonymous
AFUN015918	CYP6Z3	1209G>A	Lys403Lys	82885537	chr2	Synonymous
AFUN015918	CYP6Z3	1173G>C	Val391Val	82885573	chr2	Synonymous
AFUN015918	CYP6Z3	993G>A	Glu331Glu	82885753	chr2	Synonymous
AFUN015918	CYP6Z3	948A>G	Ser316Ser	82885798	chr2	Synonymous

AFUN015918	CYP6Z3	879A>G	Val293Val	82885867	chr2	Synonymous
AFUN015918	CYP6Z3	840A>G	Gly280Gly	82885906	chr2	Synonymous
AFUN015918	CYP6Z3	27G>T	Leu9Phe	82886719	chr2	NON_Synonymous
AFUN015919	CYP6Z1	1471C>A	Arg491Arg	82887622	chr2	Synonymous
AFUN015919	CYP6Z1	933G>A	Thr311Thr	82888222	chr2	Synonymous
AFUN015919	CYP6Z1	471_472 delinsCA	His158Asn	82888682	chr2	NON_Synonymous
AFUN015919	CYP6Z1	195C>A	Arg65Arg	82888960	chr2	Synonymous
AFUN015919	CYP6Z1	84A>G	Gly28Gly	82889071	chr2	Synonymous
AFUN015794	CYP6M2	171T>G	Val57Val	85295838	chr2	Synonymous
AFUN015794	CYP6M2	4G>T	Val2Leu	85296005	chr2	NON_Synonymous
AFUN010918	CYP6N1	735C>T	Phe245Phe	85301578	chr2	Synonymous
AFUN010918	CYP6N1	921T>C	Gly307Gly	85301764	chr2	Synonymous
AFUN010918	CYP6N1	1005T>C	Asn335Asn	85301848	chr2	Synonymous
AFUN015962	CYP6N2	450C>T	Val150Val	85316097	chr2	Synonymous
AFUN015962	CYP6N2	480A>C	Arg160Arg	85316127	chr2	Synonymous
AFUN015962	CYP6N2	491G>A	Ser164Asn	85316138	chr2	NON_Synonymous
AFUN015962	CYP6N2	1425A>G	Pro475Pro	85317133	chr2	Synonymous
AFUN015774	CYP12F4	1555G>A	Asp519Asn	87484924	chr2	NON_Synonymous
AFUN015774	CYP12F4	927G>A	Lys309Lys	87485673	chr2	Synonymous
AFUN006122	CYP4C27	732T>C	Ala244Ala	96818349	chr2	Synonymous
AFUN015907	CYP305A3	486A>G	Ser162Ser	8957858	chr3	Synonymous
AFUN015904	CYP4J5	518C>G	Ser173Cys	12809135	chr3	NON_Synonymous
AFUN001382	CYP9J5	1464T>C	Arg488Arg	73206900	chr3	Synonymous
AFUN001382	CYP9J5	1335C>T	His445His	73207029	chr3	Synonymous
AFUN001382	CYP9J5	1332T>C	His444His	73207032	chr3	Synonymous
AFUN001382	CYP9J5	1194A>C	Ser398Ser	73207170	chr3	Synonymous
AFUN001382	CYP9J5	1128A>G	Ser376Ser	73207236	chr3	Synonymous
AFUN001382	CYP9J5	864T>A	Arg288Arg	73207500	chr3	Synonymous
AFUN001382	CYP9J5	66T>C	Ile22Ile	73208849	chr3	Synonymous
AFUN001382	CYP9J5	64A>C	Ile22Leu	73208851	chr3	NON_Synonymous
AFUN001383	CYP9J5	828A>C	Val276Val	73211431	chr3	Synonymous
AFUN015862	CYP9L1	1104G>A	Val368Val	73268402	chr3	Synonymous
AFUN015862	CYP9L1	1089A>T	Glu363Asp	73268417	chr3	NON_Synonymous
AFUN015862	CYP9L1	838C>T	Leu280Leu	73268668	chr3	Synonymous
AFUN015862	CYP9L1	822A>G	Pro274Pro	73268684	chr3	Synonymous
AFUN015862	CYP9L1	774G>A	Leu258Leu	73268732	chr3	Synonymous
AFUN015862	CYP9L1	196C>T	Leu66Leu	73269379	chr3	Synonymous
AFUN015865	CYP9J4	1494T>C	Tyr498Tyr	73273890	chr3	Synonymous
AFUN015865	CYP9J4	1362T>C	Phe454Phe	73274022	chr3	Synonymous
AFUN015865	CYP9J4	1257T>C	Asp419Asp	73274127	chr3	Synonymous
AFUN015865	CYP9J4	1209A>G	Pro403Pro	73274175	chr3	Synonymous
AFUN015865	CYP9J4	1032G>T	Ser344Ser	73274352	chr3	Synonymous
AFUN015865	CYP9J4	873G>A	Met291Ile	73274511	chr3	NON_Synonymous

AFUN015865	CYP9J4	871A>G	Met291Val	73274513	chr3	NON_Synonymous
AFUN015865	CYP9J4	420A>G	Thr140Thr	73274964	chr3	Synonymous
AFUN015865	CYP9J4	417G>C	Ala139Ala	73274967	chr3	Synonymous
AFUN015865	CYP9J4	315C>T	His105His	73275069	chr3	Synonymous
AFUN015866	CYP9J3	1587T>C	Phe529Phe	73276794	chr3	Synonymous
AFUN015866	CYP9J3	1479T>G	Ala493Ala	73276902	chr3	Synonymous
AFUN015866	CYP9J3	1278G>A	Gly426Gly	73277168	chr3	Synonymous
AFUN015866	CYP9J3	1234T>G	Leu412Val	73277212	chr3	NON_Synonymous
AFUN015866	CYP9J3	912T>G	Ala304Ala	73277534	chr3	Synonymous
AFUN015866	CYP9J3	909G>A	Glu303Glu	73277537	chr3	Synonymous
AFUN015866	CYP9J3	627A>T	Glu209Asp	73277819	chr3	NON_Synonymous
AFUN015866	CYP9J3	534T>C	Gly178Gly	73277912	chr3	Synonymous
AFUN015866	CYP9J3	198A>G	Ala66Ala	73278316	chr3	Synonymous
AFUN015866	CYP9J3	118T>G	Ser40Ala	73278396	chr3	NON_Synonymous
AFUN015866	CYP9J3	99T>C	Pro33Pro	73278415	chr3	Synonymous
AFUN011481	CYP4G17	1107A>T	Gly369Gly	2975156	chrX	Synonymous
AFUN011481	CYP4G17	1005T>C	Arg335Arg	2975258	chrX	Synonymous
AFUN010428	CYP4G16	1122T>C	Ser374Ser	16590631	chrX	Synonymous
AFUN010428	CYP4G16	987T>C	Gly329Gly	16590766	chrX	Synonymous
AFUN010428	CYP4G16	738C>T	Leu246Leu	16591015	chrX	Synonymous

Table S9: Most significant SNPs per chromosome

Transcripts	Type of substitution	position	Nucleotide	Amino acid	Log10(t-test)	Gene name	Chrom	genome start position	Description
AFUN015792	SYNONYMOUS	785-785	T>C	L251L	18.5	CYP6P9a	chr2	8545397	Cytochrome P450
AFUN015792	SYNONYMOUS	743-743	C>T	L237L	21.3	CYP6P9a	chr2	8545496	Cytochrome P450
AFUN015792	SYNONYMOUS	584-584	T>G	T184T	16.3	CYP6P9a	chr2	8545598	Cytochrome P450
AFUN007146	3PRIME_UTR	1523-1523	C>T		19.6	AFUN007146	Chr3	61903247	cytochrome b5 reductase
AFUN022249	3PRIME_UTR	1147-1147	C>A		20.6	AFUN022249	Chr3	69648668	CLIP-domain serine protease
AFUN020438	3PRIME_UTR	6739-6739	T>C		21	AFUN020438	Chr3	73934894	Ecdysone-induced protein 75B
AFUN009958	3PRIME_UTR	944-944	C>A		19.6	TPX2	Chr3	81101900	Thioredoxin peroxidase 5
AFUN019973	5PRIME_UTR	118-118	T>G		20.1	AFUN019973	Chx	1587564	cytochrome c oxidase subunit 6a, mitochrondrial
AFUN019838	5PRIME_UTR	508-508	A>G		19	AFUN019838	Chrx	17083881	no description

Table S10: Genetic variability parameters of resistance genes CYP6P9a and CYP6P9b in FUMOZ and FANG from pooled RNAseq data in comparison to CYP4C36 using non-synonymous substitutions

**Kpome**	**n**	**S**	**h**	**π (k)**	**D**	**D***
			**CYP6P9a**			
**FUMOZ-R**	8	1	2	0.00035 (0.53)	1.30^{ns}	0.80^{ns}
**FANG**	8	18	8	0.0046 (7.0)	0.43^{ns}	-0.21^{ns}
**Total**	16	18	8	0.0028 (4.3)	-0.65^{ns}	-0.68^{ns}
			**CYP6P9b**			
**FUMOZ-R**	8	1	2	0.00035 (0.53)	1.30^{ns}	0.800^{ns}
**FANG**	8	16	8	0.0045 (6.9)	0.63^{ns}	0.89^{ns}
**Total**	16	18	10	0.0032 (4.9)	-0.44^{ns}	0.76^{ns}
			**CYP4C36**			
**FUMOZ-R**	8	8	5	0.0011 (1.78)	-1.06^{ns}	-0.72^{ns}
**FANG**	8	8	6	0.0018(2.8)	-0.36^{ns}	-0.19^{ns}
**Total**	16	11	10	0.0014 (2.2)	-1.36^{ns}	-1.30^{ns}

N= number of sequences (2n); S, number of polymorphic sites; π, nucleotide diversity (k= mean number of nucleotide differences); D and D* Tajima's and Fu and Li's statistics; ns, not significant
